# Thermal ablation for early-stage breast cancer: cryoablation, microwave ablation, radiofrequency ablation, high-intensity focused ultrasound ablation, and laser ablation — a systematic review

**DOI:** 10.1016/j.breast.2026.104843

**Published:** 2026-06-16

**Authors:** Judit Erdos, Julia Kern, Doris Giess, Andras Drozgyik, Gregor Goetz

**Affiliations:** a Austrian Institute for Health Technology Assessment (AIHTA), Vienna, Austria; b Faculty of Health and Sport Sciences, Széchenyi István University, Győr, Hungary

**Keywords:** Early-stage breast cancer, Thermal ablation, Cryoablation, Microwave ablation, Radiofrequency ablation, High-intensity focused ultrasound ablation, Laser ablation

## Abstract

**Background:**

Thermal ablation techniques have been proposed as minimally invasive alternatives to surgery for early-stage breast cancer (BC), with the potential to reduce treatment burden while preserving oncological outcomes. The aim of this review was to evaluate the available evidence on the effectiveness and safety of thermal ablation techniques in patients with early-stage BC.

**Methods:**

We conducted a systematic review and narrative synthesis in accordance with PRISMA guidelines. MEDLINE, Embase, the Cochrane Library, and the INAHTA database were searched for studies published between 2014 January and 2026 May. Eligible studies included randomised controlled trials, non-randomised comparative studies, and prospective single-arm studies enrolling adult patients with early-stage BC treated with cryoablation (CYA), microwave ablation (MWA), radiofrequency ablation (RFA), high-intensity focused ultrasound ablation (HIFU), or laser ablation (LA), with or without standard care. Risk of bias was assessed using RoB 2.0 and ROBINS-I, and certainty of evidence was evaluated using GRADE.

**Results:**

Twenty-two trials (in 24 publications) were included: twelve on CYA, five on MWA, three on RFA, two on HIFU, and one on LA, with one trial evaluating both CYA and MWA. Comparative evidence was available for CYA, MWA, and RFA, while HIFU and LA were evaluated exclusively in single-arm studies. Across interventions, recurrence rates were generally low, overall survival was high, reported adverse events were predominantly mild to moderate. Most studies were small, had short follow-up, and frequently included post-ablation surgical resection, limiting causal attribution. Certainty of evidence ranged from very low to moderate, depending on intervention and outcome.

**Conclusion:**

Thermal ablation techniques demonstrate favourable short-term safety profiles and promising oncological outcomes in selected patients with early-stage BC. Current evidence is limited by low certainty, heterogeneous study methodologies, and limited long-term comparative data. High-quality comparative studies with standardised assessment methods and long-term follow-up are required to elucidate the comparative clinical evidence of thermal ablation techniques and inform their role in routine clinical practice.

## Highlights

• Five thermal ablation techniques were assessed for early-stage breast cancer.
• Comparative evidence was available only for three ablation techniques.
• Overall survival and recurrence outcomes were generally favourable, with mostly mild to moderate adverse events.
• Most studies were small, non-comparative, and had limited follow-up.
• Certainty of evidence ranged from very low to moderate.

## Graphical abstract

**Figure:**
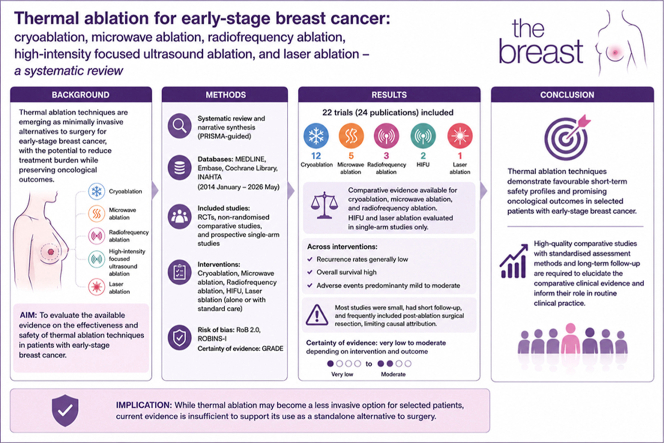

## 1 Introduction

Breast cancer (BC) is the most common cancer among women worldwide and the second most common cancer overall [1]. Advances in screening technologies over the last two decades have significantly improved the early detection of BC, especially in high-income countries [2]. Early-stage diagnosis is critical in BC, as tumour size (T stage) is one of the most important prognostic factors, and detecting the disease early is typically associated with favourable outcomes, with reported five-year survival rates of at least 90% [3,4]. Patients with early-stage BC undergoing breast-conserving treatment have low reported local recurrence rates, with recent studies reporting five-year cumulative incidences ranging from below 1% to approximately 5% [5,6]. While surgery remains the gold standard for treating early-stage BC, there is a notable progression towards less invasive methods, reflecting a broader trend in oncological practices aimed at reducing patient burden while maintaining high efficacy [7,8]. A prominent example of this is axillary surgery; in line with the 2025 St. Gallen Breast Cancer Consensus and decades of de-escalation efforts, the indication for axillary lymph node dissection continues to narrow, with regional irradiation increasingly used as an alternative [9].

The evolution from radical mastectomy to breast-conserving surgery (BCS) exemplifies this shift towards less invasive procedures. Building on this trend, various thermal ablation therapies such as radiofrequency ablation (RFA), microwave ablation (MWA), cryoablation (CYA), high-intensity focused ultrasound ablation (HIFU), and laser ablation (LA) are being explored as possible alternatives to surgical resection for treating early-stage BC [10]. Although established for thyroid, liver, and lung cancers [[11], [12], [13], [14], [15], [16], [17], [18], [19]], their use in BC remains experimental. Regulatory approvals currently cover benign and malignant soft tissue tumours and benign breast lesions; however, they are not specifically approved to treat BC [8]. Consequently, these techniques are not yet included in standard BC treatment guidelines, reflecting the ongoing need for robust clinical evidence to support their efficacy and safety.

Currently, most thermal ablation interventions for BC are proposed for tumours smaller than 5 cm, with a particular focus on tumours no larger than 2 cm due to the limited treatment radius within which complete cell death can be ensured [7,8,20]. The claimed benefits of these techniques include lower anaesthesia requirements, quicker recovery, and improved cosmetic outcomes compared to more invasive methods [8].

Previous systematic reviews and meta-analyses have evaluated thermal ablation techniques for breast cancer [[21], [22], [23], [24], [25], [26], [27], [28]]. However, earlier reviews either included predominantly older studies, focused primarily on technical feasibility, or restricted inclusion to selected tumour sizes and ablation modalities. Since then, additional prospective comparative and non-comparative studies with longer follow-up have become available, including studies evaluating thermal ablation as definitive treatment without routine surgical resection. Therefore, an updated synthesis of the current evidence on the safety and efficacy of thermal ablation interventions for early-stage BC is warranted.

This systematic review aims to assess the safety and efficacy of thermal ablation interventions alone or in conjunction with standard care compared to standard care alone in patients with early-stage BC, with respect to mortality, complete ablation rate, residual tumour rate, recurrence, and (serious) adverse events (AEs).

## 2 Methods and materials

### 2.1 Search strategies

**Fig. 1:**
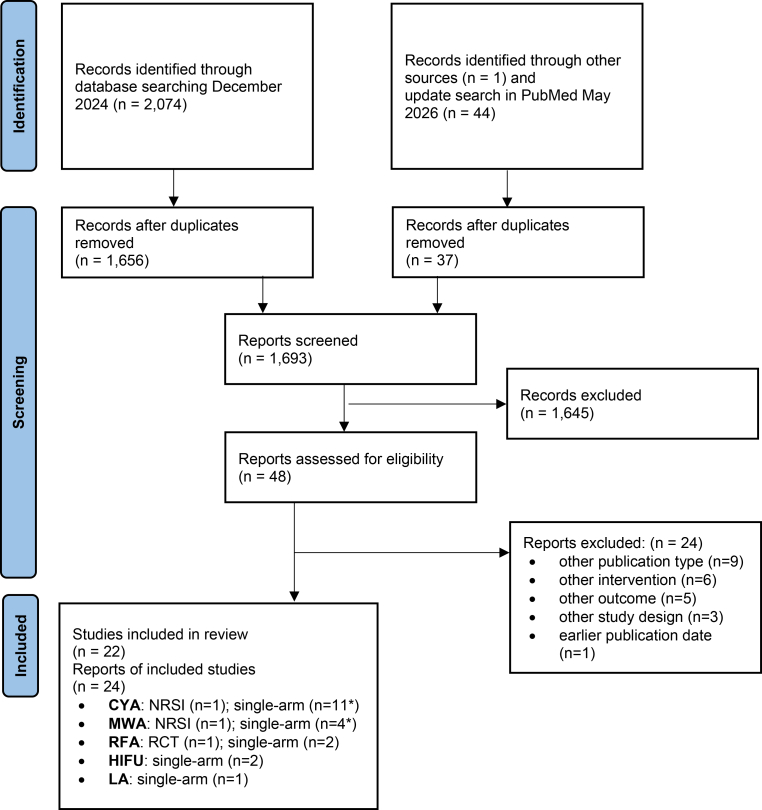
Flow chart of study selection (PRISMA Flow Diagram) Abbreviations: CYA – cryoablation, HIFU – high-intensity focused ultrasound ablation, LA – laser ablation, MWA – microwave ablation, NRSI – non-randomised study of intervention, RFA – radiofrequency ablation.

This review adhered to the Preferred Reporting Items for Systematic Reviews and Meta-Analyses (PRISMA) guidelines (Fig. 1). A systematic literature search was conducted in December 2024 in the following databases: Medline via Ovid, Embase, the Cochrane Library and the International Network of Agencies for Health Technology Assessment database. The search spanned from 2014 to 2024, and in Medline and Embase to articles published in English or German. An update search was conducted in PubMed in May 2026 using the original search strategy to identify studies published between December 2024 and May 2026. The detailed search strategies for each database are provided in Supplementary material B. The study protocol was registered on the Open Science Framework platform [29].

### 2.2 Study selection

Studies were eligible if they met the following criteria: inclusion of adult patients (≥18 years) with early-stage BC, treated with thermal ablation using any of the following interventions: RFA, HIFU, MWA, CYA, LA with or without resection and, if applicable, with adjuvant therapy (e.g. radiotherapy, chemotherapy, endocrine therapy, and immunotherapy). The outcomes of interest were centred on survival and disease control, encompassing overall (OS) and disease-specific survival (progression-free or disease-free survival (DFS)), recurrence (local and distant), complete ablation rate, quality of life, cosmetic results and any safety outcomes (serious and non-serious). Eligible studies were systematic reviews, randomized controlled trials (RCTs), non-randomized comparative studies and non-comparative prospective studies with a minimum of 10 patients.

The exclusion criteria encompassed studies in languages other than English or German, those published outside the period, retrospective studies, case reports, case series with fewer than 10 patients, conference abstracts, editorials, letters, study protocols, and publications without full-text results, or those failing to report on the predefined endpoints of interest. Studies evaluating thermal ablation in patients with benign tumours, such as fibroadenoma, metastatic tumours or recurrent disease were excluded. Studies were also excluded if thermal ablation was applied after standard therapy, e.g. for cancer-related pain management.

After removing duplicates, two researchers independently screened titles, abstracts, and full-text articles for eligibility, extracted the data, and assessed the risk of bias and the certainty of the evidence. Disagreements were resolved by consensus. We also examined references from included publications and relevant review articles to ensure comprehensive coverage of the literature.

### 2.3 Data extraction

The data was extracted into a data extraction form on Excel, containing the following variables: study details (author, year, setting, design, funding), patient inclusion and exclusion criteria, patient demographics, tumour details (size, histology, hormone receptor status), treatment details, and outcomes. The data were extracted by one researcher and verified by a second researcher. Outcome data were extracted as reported by the original studies, including proportions, medians, means, confidence intervals, and recurrence rates where available.

### 2.4 Data analysis and synthesis

We classified studies according to our predefined PICO. RCTs that did not include a comparator relevant to the predefined PICO (e.g., dose-escalation or modality-selection designs) were analysed descriptively as uncontrolled (single-arm) studies. Due to substantial heterogeneity in study design, patient selection, ablation modalities, outcome definitions, and follow-up duration, a quantitative meta-analysis was not performed. Findings were synthesised narratively.

Risk of bias assessment was performed independently by two researchers using the Cochrane Risk of Bias 2.0 Tool [30] for RCTs and for non-randomised studies, the Risk of Bias in Non-Randomised Studies of Interventions (ROBINS-I) [31]. Single-arm trials were classified as having a high risk of bias according to methodological guidance by the HTA Coordination Group pursuant to the HTA Regulation [32], and as a result, they were not subject to further assessment.

The certainty of evidence was assessed with the Grading of Recommendations, Assessment, Development, and Evaluation (GRADE) framework by summarizing all relevant study results for each endpoint and subsequently assessing the certainty of the evidence.

## 3 Results

The systematic search in databases identified, in total, over 2000 publications on the five thermal ablation interventions. After removing duplicates, 1656 publications were screened, and 44 full-text articles were obtained and assessed for eligibility. The update search identified 37 additional records published between December 2024 and May 2026. After screening, three full-text articles were assessed for eligibility. Our final pool of relevant studies comprised 11 studies for CYA, five for MWA, three for RFA, two for HIFU and one for LA. A list of excluded studies with the reason for exclusion is available in the Supplementary material B.

### 3.1 Cryoablation

#### 3.1.1 Study characteristics

Twelve studies were included (in 14 publications), one case-control [11,33] single-arm studies [[34], [35], [36], [37], [38], [39], [40], [41], [42], [43], [44], [45], [46]]. The case-control [33] study was multicentre, conducted in Italy, and sponsored by various European medical organizations. The study assessed CYA against standard surgery and included 20 patients with solitary invasive BC ≤ 2 cm, ten in both study arms. Both groups had similar mean ages. The follow-up lasted up to 21 days when the tumour resection was performed in the CYA group.

The single-arm trials [[34], [35], [36], [37], [38], [39], [40], [41], [42], [43], [44], [45], [46]] were conducted across China [40], France [34], Japan [38], Spain [42], the Netherlands [45,46], and the USA [[35], [36], [37],^39^,^41^,[43], [44], [45], [46]], involving a total of 562patients. Of these, five were multicentric [35,36,41,[43], [44], [45], [46]], five were conducted at a single centre [34,[38], [39], [40],^42^], and one did not specify its setting [37]. The studies were variably funded, including support from university hospitals, medical device companies, and other grants. Tumour size inclusion criteria varied across the trials, ranging from ≤1.5 cm to ≤3 cm. Follow-up durations ranged from 3 to 73 months, with six studies [33,[40], [41], [42], [43], [44]] performing post-ablation tumour resection to confirm complete ablation histopathologically, with assessment conducted from immediately post-intervention to three months after treatment. Six other studies [[34], [35], [36], [37], [38], [39],^44^] did not perform routine resection; in three of these studies, complete ablation was assessed using imaging with or without histopathological evaluation, one study used core needle biopsy and two did not report this outcome. Further details are available in the Supplementary material A (Tables A-1 to Table A-5).

#### 3.1.2 Quality of the included studies (risk of bias)

The only outcome with comparative data was AEs in the case-control study [33] and it was judged to have a critical risk of bias due to confounding. For other outcomes in this study [33], as well as for all outcomes in the single-arm studies [[34], [35], [36], [37], [38], [39], [40], [41], [42], [43], [44], [45], [46]], the risk of bias was judged to be high. Further details are available in the Supplementary material A (Risk of bias tables).

#### 3.1.3 Efficacy results

Ten single-arm trials [[34], [35], [36], [37], [38], [39], [40], [41],[43], [44], [45], [46]] (n = 503) reported OS, of which six [34,[39], [40], [41],^43^,^46^] (n = 194) reported a follow-up ranging from one to 12 months, and four [[35], [36], [37], [38],^44^] (n = 307) a follow-up of 12 to 73 months. The studies with the shorter-term follow-up reported a 99% OS rate. Two deaths were noted, one due to a myocardial infarction [34] and the other to undefined unrelated cause [39]. The studies with the longer follow-up reported a 92% OS rate. All reported deaths occurred in two trials [36,44]. Most deaths were unrelated to BC or due to unknown causes [36,44], whereas two deaths were attributed to distant metastasis [36]. DFS was not reported in any of the studies. Complete ablation was reported in the case-control study [33] for the CYA group (n = 10) and in seven single-arm studies [37,[40], [41], [42], [43], [44],^46^] (n = 295) and was achieved in 92% of participants (range: 53% to 99%). Lower complete ablation rates were observed in studies with later post-treatment assessment. Recurrence was reported in six single-arm trials [34,[36], [37], [38], [39],^44^] (n = 364) with 20 recurrences noted (5%, range: 0 to 22%) over 18 to 60 months of follow-up.

#### 3.1.4 Safety results

No SAEs were noted in the case-control study [33] (n = 40), as well as in the five single-arm trials [34,[36], [37], [38],^46^] (n = 265) that measured this outcome. The follow-up periods of these studies spanned from two to 60 months. Regarding AEs, the case-control study [33] reported two minor incidents, specifically post-ablative hematomas, both occurring in the intervention group and resolving by the one-week follow-up. The same study [33] recorded lower pain scores in the intervention group compared to the control group. Five single-arm trials [34,37,[40], [41], [42]] (n = 129) reported AEs immediately following the procedure, predominantly mild to moderate pain and bruising. The proportion of patients experiencing AEs varied substantially across studies, ranging from 0% to 100%. Five single-arm studies [34,37,38,41,44] (n = 158) reported AEs with follow-up periods ranging from one week to six months. Two of them reported the number of events rather than affected patients, while the remaining three reported that 0% to 6% of patients experienced AEs. Reported events were predominantly mild (Grade 1–2). One single-arm study [36] (n = 194) with five-year follow-up did not specify when the AEs were recorded. It reported that AEs occurred in 39% of patients during the follow-up, the majority of which were mild or moderate.

#### 3.1.5 Certainty of the evidence (GRADE)

The certainty of evidence was assessed as low for recurrence and complete ablation when considering evidence from single-arm studies, while all other outcomes, including overall survival and safety outcomes, were rated as very low. Details for each outcome are presented in the Evidence Profile tables in the Supplementary material A.

### 3.2 Microwave ablation

#### 3.2.1 Study characteristics

Five studies were included, one prospective propensity-score-matched study [47] and four single-arm trials [45,46,[48], [49], [50]]. The propensity-score-matched study [47] was multicentre, conducted in China, and sponsored by research grants. It included 132 patients who were all over 70 years old and had tumours ≤3 cm in size. Of these, 33 were treated with MWA in conjunction with endocrine therapy and 99 underwent standard surgery with standard adjuvant therapies. The median follow-up was 31 months. In the MWA group, tumour ablation was assessed through imaging without subsequent resection.

The four single-arm trials [45,46,[48], [49], [50]] included a total of 119 patients with maximum tumour sizes ranging from ≤2 cm to ≤3 cm, and mean ages ranging from 50 to 66 years. One of these trials [49] was initially designed as a multi-arm RCT; however, only the two arms involving MWA—one with subsequent resection and the other without—were included in our analysis. The two MWA-arms were analysed as a single cohort. Another trial [45,46] was also designed as a multi-arm RCT but only the MWA arm was relevant for our analysis and was included as a single cohort. Three single-arm studies [[48], [49], [50]] were conducted in China and one [45,46] in the Netherlands. Across studies, complete ablation was predominantly assessed using histopathological confirmation after surgical excision, with assessment performed from immediately post-intervention to three months after treatment. One study [50] additionally included an MWA-only arm in which complete ablation was evaluated using imaging one week after treatment. Further details are available in the Supplementary material A (Tables A-6 and A-7).

#### 3.2.2 Quality of the included studies (risk of bias)

The propensity-score-matched study [47] was judged to have a serious risk of bias due to confounding and missing data concerns for the outcomes OS, DFS and recurrence. For all outcomes in the single-arm studies [[48], [49], [50]], the risk of bias was judged to be high. Further details are available in the Supplementary material A (Risk of bias tables).

#### 3.2.3 Efficacy results

In the propensity-score-matched study [47] (n = 132), at a median follow-up of 31 months, the OS was 100% in the intervention group and 99% in the control group (hazard ratio [HR] 0.537; 95% CI: 0.089-3.325, p = 0.49). The one-year OS rate was 97% versus 100% and the three-year OS rate 93% versus 96%. No deaths were reported in any of the four single-arm trials [46,[48], [49], [50]] (n = 119) during follow-ups ranging from immediately post-intervention to 36 months. Only the propensity-score-matched study [47] reported tumour progression and recurrence rates over a median follow-up of a 31 months. Tumour progression was experienced by 3% of patients in both study arms (HR 0.536; 95% CI: 0.128–2.249, p = 0.38). Local recurrence rates were 3% in the MWA group and 1% in the control group, while distant recurrence was 0% in the MWA group and 2% in the control group. Additionally, complete ablation assessed by imaging was reported in 97% of patients undergoing MWA in this study [47]. The single-arm trials [46,[48], [49], [50]] reported complete ablation rates ranging from 72 to 100%, with lower rates observed in the study assessing complete ablation histopathologically three months after MWA. Studies assessing complete ablation immediately or one week after treatment using histopathological and/or imaging-based methods reported rates ranging from 85% to 100%. Of the single-arm trials, only the one with two MWA cohorts [49] (n = 35) reported on recurrence, noting no cases within a follow-up period of 13 to 47 months in patients who received MWA without subsequent resection. No data on recurrence was available for patients who underwent resection after MWA [49].

#### 3.2.4 Safety results

SAEs were not reported in any of the studies. In the propensity-score-matched study [47], there were no AEs in the MWA group, but AEs were not recorded for the control group. Among the single-arm trials, three [[48], [49], [50]] (n = 101) reported pain, affecting 6% to 16% of patients. Two studies [45,46,49] (n = 58) reported local skin burns in 10% and 33% of patients and local skin necrosis in 2.5%. Two trials [48,50] (n = 61) reported swelling, with incidence rates ranging from 42% to 100%.

#### 3.2.5 Certainty of the evidence (GRADE)

All outcomes assessed were rated as very low certainty. The Evidence Profile table in the Supplementary material A presents the outcome-specific certainty assessments.

### 3.3 Radiofrequency ablation

#### 3.3.1 Study characteristics

Three studies were included, one RCT [51] and two single-arm trials [52,53]. The RCT [51] was an open-label, single-centre study conducted in Spain. No information was provided on funding. The study compared RFA followed by immediate resection with histopathological assessment of complete ablation to lumpectomy alone, involving 20 women per study arm, with a mean age of 64 years and tumour sizes ≤2 cm. A statistically significantly higher proportion of patients in the RFA group received breast irradiation compared to the control group (p = 0.038); no other differences were observed between the groups. The median follow-up time was 26 months for the RFA group and 24 months for the lumpectomy group.

Two single-arm trials [52,53] were conducted in Sweden and Japan, and included 18 and 370 patients, respectively. Both studies enrolled women with unifocal early-stage BC and restricted inclusion to tumours ≤2 cm [52] or ≤1.5 cm [53], respectively, with predominantly hormone receptor-positive disease. Study designs differed substantially regarding post-ablation management. In the Swedish study [52], all patients underwent subsequent surgical resection after ablation, whereas the Japanese study [53] used RFA as definitive treatment without routine surgical resection, except in cases with suspected residual tumour on biopsy or imaging. Adjuvant radiotherapy and endocrine therapy were commonly administered in both studies. Follow-up duration ranged from a median of 14.5 days in the Swedish study to 5 years in the Japanese study. Further details are available in the Supplementary material A (Table A-8 and A-9).

#### 3.3.2 Quality of the included studies (risk of bias)

The included RCT [51] was judged to have a low risk of bias for the following outcomes: mortality, complete tumour ablation, recurrence and safety. For all outcomes in the single-arm studies [52,53], the risk of bias was judged to be high. Further details are available in the Supplementary material A (Risk of bias tables).

#### 3.3.3 Efficacy results

OS was reported in the RCT [51] (n = 40), with 100% in both the RFA with subsequent resection and BCS arms at a median follow-up of 25 months. In the single-arm trial with five-year follow-up (n = 370) [53], OS was 99.2% (95% CI 97.4–99.7). Four deaths occurred, of which two were related to BC. In the single-arm trial [52] (n = 18) with 14-day follow-up, all patients were alive at the assessed timepoint. DFS was not reported in any of the studies. The RCT [51] (n = 20) and the single-arm trial [52] (n = 18) with short-term follow-up, both reported 100% complete tumour ablation in the intervention groups. The single-arm trial [53] with the longer-term follow-up reported 97% complete ablation at three months. No recurrence of local or distant tumours was observed in the RCT [51] (n = 40) during a median follow-up of 25 months. In the single-arm trial [53] reporting recurrence outcomes, the five-year cumulative ipsilateral breast tumour recurrence rate was 0.6% (95% CI 0.1–1.9).

#### 3.3.4 Safety results

Serious adverse events were not reported as a separate outcome category in the RCT [51] and in one of the single-arm studies (n = 18) [52]. In the other single-arm study (n = 370) [53], one Grade 3 skin ulceration (0.3%) and no Grade 4 events were reported. In the RCT [51] (n = 40), postoperative local AEs occurred in 40% of patients in the RFA group versus 5% in the BCS group (p = 0.1). Brest inflammation was reported in 25% versus 5% of patients, while breast infections occurred in 15% versus 0% of patients in the RFA and BCS groups, respectively; these differences were not statistically significant (p = 0.18 and p = 0.23). The study was terminated early due to the higher number of AEs in the RFA study arm. In one single-arm trial [52] (n = 18), pain was the only AE reported, assessed using the visual analogue scale. On a 10-point scale, median pain scores were 2 during local anaesthetic administration and 2.5 during the procedure, decreasing to 0.5 post-procedure. No other AEs were noted in this trial. In the other single-arm trial (n = 370) [53] seven Grade 1–2 thermal burns were reported during RFA. Between RFA and the initiation of radiotherapy, 17 Grade 1 and one Grade 2 AE were reported.

#### 3.3.5 Certainty of evidence (GRADE)

Evidence from the RCT was rated as low certainty for overall survival, complete ablation, and recurrence, and as moderate certainty for adverse events. Evidence from the single-arm study was rated as very low certainty overall. The Evidence Profile table in the Supplementary material A presents the outcome-specific certainty assessments.

### 3.4 High-intensity focused ultrasound ablation

#### 3.4.1 Study characteristics

Two single-arm trials were included [54,55]. One trial [54], was originally designed as RCT, comparing HIFU plus mastectomy to mastectomy alone, but all relevant outcomes were assessed only after both arms underwent mastectomy. Because the co-intervention could account for any between-group differences, the causal effect of HIFU could not be isolated. In line with our prespecified approach, we therefore did not use the randomised comparison and extracted single-arm data from the HIFU arm only. This arm included 25 women aged 22-65 years with tumour size ≤5 cm. was analysed as a single-arm trial. Complete ablation was assessed histopathologically after resection within one to two weeks following treatment.

Another study, a prospective case series [55] conducted in the Netherlands and sponsored by research funds, involved 10 female patients with an average age of 55 years. Patients underwent HIFU followed by lumpectomy, except for one patient who required no further surgery. This study focused on tumour sizes ≤1 cm with a follow-up ranging from 48 h to 10 days. Complete ablation was not assessed. Further details are available in the Supplementary material A (Table A-10).

#### 3.4.2 Quality of the included studies (risk of bias)

The risk of bias in both studies [54,55] was judged to be high for all outcomes. Further details are available in the Supplementary material A (Risk of bias tables).

#### 3.4.3 Efficacy results

OS was 100% in both studies [54,55] (n = 35), with follow-up periods of 10 days and 12 months post-procedure. DFS was not reported in neither study. One trial [54] (n = 25) reported complete tumour ablation in all participants and no recurrences within 12 months, whilst the other [55] did not report on these outcomes.

#### 3.4.4 Safety results

SAEs were not reported in the trials. However, mild AEs were reported in both [54,55] (n = 35). The trial with the longer follow-up [54] (n = 25) reported oedema in all patients, pain in 44%, and mild fever in 12% at 12 months. The trial with the 10-day follow-up [55] (n = 10) reported five minor AEs, including nausea and vomiting, moderate pain (pain score 4 and 5 on a scale of 10) and skin changes.

#### 3.4.5 Certainty of evidence (GRADE)

All outcomes assessed were rated as very low certainty. The Evidence Profile table in the Supplementary material A presents the outcome-specific certainty assessments.

### 3.5 Laser ablation

#### 3.5.1 Study characteristics

One single-arm trial was included [56]. The trial was manufacturer-sponsored and was conducted in the USA and UK. The study included 61 female patients aged 42 to 77 years. Most patients (90%) underwent a lumpectomy within 28 days post-ablation. Complete ablation was assessed histologically after resection The inclusion criteria excluded patients with neoadjuvant treatments and allowed only those with tumour size ≤2 cm, minimal intraductal components, and no significant comorbidities. Follow-up ranged up to five years, with a mean duration of 43 months. Further details are available in the Supplementary material A (Table A-11).

#### 3.5.2 Quality of the included studies (risk of bias)

The risk of bias was judged to be high for all outcomes [56]. Further details are available in the Supplementary material A (Risk of bias tables).

#### 3.5.3 Efficacy results

OS and DFS were not reported in the trial [56]. Complete tumour ablation, confirmed histologically, was achieved in 84% of patients. Recurrence rate was 3% at four years.

#### 3.5.4 Safety results

The trial [56] reported eight mild and six moderate AEs at 43 months. The moderate events included pain in four cases, one lump and one seroma. The mean pain score was 4.2 from a maximum of 10. SAEs were not reported.

#### 3.5.5 Certainty of evidence (GRADE)

All outcomes assessed were rated as very low certainty. The Evidence Profile table in the Supplementary material A presents the outcome-specific certainty assessments.

## 4 Discussion

Our review synthesised evidence on five thermal ablation techniques versus surgery, with or without adjuvant therapy, in patients with early-stage BC, focusing on prespecified patient-relevant outcomes. Comparative data were available for CYA, RFA and MWA.

In summary, all five interventions demonstrated a favourable safety profile both short and the long-term. Complete ablation rates generally high across modalities, frequently exceeding 90%, although one MWA study reported a lower complete ablation rate of 72%, which may partly reflect differences in timing and methods of post-ablation assessment. Recurrence rates were generally low, and when OS and DFS rates were reported, they were high and comparable to the control group. A summary of key findings by ablation type is presented in Table 1.

**Table 1:** Summary of findings for the thermal ablation therapies: cryoablation, microwave ablation, radiofrequency ablation, high intensity focused ultrasound ablation and laser ablation in early-stage breast cancer patients.

Intervention	CYA	MWA	RFA	HIFU	LA
Included studies (number, type, included total population)	12 studies: 1 case-control (CYA: n = 20 vs surgery: n = 20), 11 single-arm (n = 562)	5 studies: 1 propensity-score-matched (MWA: n = 33 vs surgery: n = 99), 4 single-arm (n = 119)	3 studies: 1 RCT (RFA: n = 20 vs surgery: n = 20), 2 single-arm (n = 388)	2 single-arm (n = 35)	1 single-arm (n = 61)
Efficacy findings	**OS** *5 single-arm, n=194* 1 - 12 m: 99% *3 single-arm, n=309* 12 - 73 m: 92% **Complete ablation** *CYA-arm of the case-control study and 7 single-arm, n=293* 92% (median 92%; range: 53% to 99%) **Recurrence** *6 single-arm, n=364* 18 m – 60 m: 5% (median 3%; range: 0 to 22%)	**OS** *MWA vs surgery, n=132* Median 31 m: 100% vs 99% (HR 0.537; 95%CI: 0.089-3.325, p = 0.49) 1 y: 97% vs 100% 3 y: 93% vs 96% *4 single-arm, n=119* <36 m: 100% **DFS** *MWA vs surgery, n=132* Median 31 m: HR 0.563; 95% CI: 0.128-2.240, p = 0.38 (in 3% vs 3% tumour progression) **Complete ablation** *MWA-arm in the propensity-score-matched study (n=33)* 1 w: 97% (1 patient required re-treatment) 1 m: 100%, 95% CI: 89.4-100% *4 single-arm, n=119* 10 days: 94% 3 m: 72% **Recurrence** *MWA vs surgery, n=132* Local Median 31 m: 3% vs 1% Distant Median 31 m: 0% vs 2% *1 single-arm, n=35* Median 13-47 m: 0%	**OS** *RFA vs surgery, n=40* 25 m: 100% vs 100% *2 single-arm, n=388* 2 w: 100% 5 yrs: 99% **Complete ablation** *RFA-arm of the RCT, 2 single-arm, n=408* Immediate post-RFA, histology: 100% 2 w, MRI: 100% 2 w, histology: 83% - 85% 3 m, histology: 97% **Recurrence** *RFA vs surgery, n=40* Local 25 m: 0% vs 0% Distant 25 m: 0% vs 0% *1 single-arm, n=370* 5 yrs: cumulative ipsilateral breast tumour recurrence 0.6% (95% CI 0.1–1.9%)	**OS** *1 study, n=10* 10 d: 100% **Complete ablation** *1 study, n=25* 100% **Recurrence** *1 study, n=25* 12 m: 0%	**Complete ablation** *1 study, n=61* 84% **Recurrence** *1 study, n=61* 48 m: 3%
Safety findings	**AE** *CYA vs surgery, n=40* <1 w: 2% vs 0% 1 w: 0% vs 0% *5 single-arm, n=129* <1 w: 30% (median 22%; range: 0 to 100%) *8 single-arm, n=352* 1 w - 5 y: predominantly mild/moderate AEs; studies variably reported event numbers and affected patient numbers (patients: median 6%; range: 0 to 50%; events: range 43 to 187) **SAE** *CYA vs surgery, n=40* 1 w: 0% vs 0% *5 single-arm, n=265* 2 m – 60 m: 0%	**AE** *MWA-arm in the propensity-score matched study, 3 single-arm, n=33* 0 events *4 single-arm, n=119* <36 m: most commonly pain (6-18%), swelling (42-100%), skin burns (10-33%)	**AE** *RFA vs surgery, n=40* Total: 40% vs 5%, p = 0.1 Breast inflammation: 25% vs 5%, p = 0.18 Breast infection: 15% vs 0, p = 0.23 *2 single-arm, n=388* Minor pain, minor thermal burn	**AE** *1 study, n=10* 10 d: minor AEs *1 study, n=25* 12 m: oedema (100%), pain (44%), mild fever (12%)	**AE** *1 study, n=61* 43 m: mild (8 events), moderate (6 events)
Conclusion	Very low and low certainty evidence indicates no negative effects on OS and recurrence on the long-term, over 90% complete ablation rates and minor safety events.	Very low certainty evidence indicates comparable OS, DFS, recurrence on the long-term and generally high, over 90% complete ablation rates, as well as good safety profile on the long-term.	Very low and low certainty evidence indicates comparable OS, no recurrence, 83% to 100% complete ablation, for AEs evidence is moderate but the RCT was terminated early due to higher AE rate in the RFA-group.	No comparative evidence, very low certainty evidence indicates no negative effects on OS, however with extremely short follow-up, no recurrence, 100% complete ablation and only minor AEs.	No comparative evidence, very low certainty evidence indicates LA is safe and the recurrence rate is low on the long-term.

Abbreviations: AE – adverse events, CYA – cryoablation, d – days, DFS – disease-free survival, HIFU – high-intensity focused ultrasound, HR – hazard ratio, LA – laser ablation, m – months, n – number, MWA – microwave ablation, RCT – randomized controlled trial, RFA – radiofrequency ablation, OS – overall survival.

Our findings align with recent systematic reviews [21,22,25,27,28] but extend the evidence base by including newly published comparative studies for CYA, RFA, and MWA—interventions for which the evidence has evolved modestly over the past decade. In contrast, comparative evidence remains absent for HIFU and LA. Our review also reports patient-relevant outcomes for HIFU, whereas previous reviews focused only on histopathological, immunological, and vascular effects. The most recent systematic review on LA [22] recommended restricting use to clinical trials pending more robust evidence. Although all earlier reviews judged the overall evidence base limited, they consistently found favourable safety and recurrence outcomes for CYA, high complete-ablation rates with acceptable safety for RFA, and no major safety concerns for HIFU.

### 4.1 Strengths and limitations of the review and the included evidence

We reported findings using the GRADE approach [57] for a transparent and structured certainty assessment and excluded retrospective studies to further improve the overall methodological rigour of our review. Unlike many previous reviews that primarily concentrated on technical feasibility or biophysical endpoints, our analysis prespecified patient-relevant outcomes, such as OS, DFS, local and distant recurrence and adverse events, thereby enhancing the clinical relevance of our findings.

This review has some limitations. The literature search was limited to the past decade to focus on contemporary clinical practice and currently used ablation protocols and devices. Nevertheless, earlier studies may still provide relevant information, particularly as some thermal ablation techniques and treatment principles have remained relatively stable over time. The update search was conducted in PubMed only and did not include all databases searched in the original review. Consequently, some recently published studies indexed exclusively in other databases may not have been identified. Additionally, we limited our sources to peer-reviewed articles in English or German, potentially overlooking relevant grey literature and studies in other languages. However, empirical evidence suggests that language restrictions are associated with a low risk of materially affecting effect estimates or review conclusions [58,59].

The available evidence has several limitations. Most studies are single-arm trials with small sample sizes and short follow-up, limiting generalisability and precluding robust comparisons with surgery. High-quality RCTs and non-randomised comparative studies are scarce. The only available RCT was underpowered due to premature complications-related termination during recruitment. Non-randomised comparative studies were generally at high risk of confounding. Furthermore, patient-relevant outcomes, particularly DFS, were often underreported, and short follow-up periods hindered the assessment of long-term outcomes, such as mortality and disease-specific recurrence.

Confounding factors related to post-ablation resection (15 of 22 studies, typically within eight weeks) limits the ability to isolate long-term oncological outcomes attributable specifically to thermal ablation alone. The varying reporting of adjuvant treatments, particularly radiation, further complicates interpretation, as these treatments likely influence local control and recurrence rates.

Additionally, studies assessing the same thermal ablation technology applied heterogeneous protocols, especially in device application and procedural standardisation. Determination of complete ablation differed between studies, with some using post-ablation surgical resection for histopathological confirmation, while others relied on imaging and biopsy-based follow-up strategies in the absence of routine surgical excision. Furthermore, the reporting of patient-relevant outcomes was also heterogeneous across studies. Health-related quality of life and cosmetic outcomes were only reported in a few studies and often in a variety of ways. The vast majority of studies did not report which adverse event grading framework was used (e.g., CTCAE), nor did they provide definitions for severity levels such as “mild” and “moderate,” limiting interpretability and comparability across studies.

### 4.2 Challenges with thermal ablation technologies and considerations/directions for future research

BC subtypes differ significantly in morphology, posing specific challenges for ablative, image-guided interventions [60]. Unlike conventional surgery, where resection margins can be adapted to tumour shape, thermal ablation relies on creating a roughly spherical zone of necrosis. This makes complete destruction more difficult in irregular or diffusely infiltrating lesions. Tissue density and thermal conductivity also impact the effectiveness of ablation, making these factors essential when choosing the appropriate treatment strategy [61]. For example, ductal carcinomas often have stellate extensions and intraductal spread, complicating targeting, while lobular carcinomas infiltrate diffusely due to E-cadherin deficiency, challenging both radiological and procedural standardisation [62].

A major challenge with thermal ablation remains the verification of complete ablation, particularly in studies without routine surgical resection. In non-resection settings, interpretation of post-ablation imaging may remain challenging, especially early after treatment, as treatment-related changes such as fat necrosis can overlap with suspicious imaging findings [8,63]. Although MRI- and biopsy-based follow-up strategies show promising accuracy in selected patients [8,43,46], available evidence does not yet support imaging as a complete substitute for histopathological confirmation, and assessment methods remain heterogeneous across thermal ablation modalities and study protocols [8,43,46,63]. It should also be acknowledged that absence of microscopic residual disease cannot be guaranteed even following breast-conserving surgery. These concerns are increasingly recognized by experts as critical factors in determining future clinical indications for thermal ablation interventions [8]. An expert panel recommended thermal ablation without resection only for well-defined, ≤1 cm, axillary-negative, luminal A-like tumours, excluding invasive lobular cancers. They suggest that broader use should occur only after confirming the effectiveness and safety of the technique in these cases [10].

Despite promising results, substantial gaps remain for the clinical use of thermal ablation for BC treatment. High-quality, long-term RCTs and non-randomised comparative studies are needed to close these gaps in the routine clinical use. A non-inferiority study design would be appropriate to demonstrate whether these minimally invasive technologies are (at least) as effective and safe as the standard intervention, surgical excision, which is the most invasive treatment option.

A search of clinical trial registries identified only one ongoing RCT (CYA versus lumpectomy in patients with T1-stage BC, estimated completion date 2034), two non-randomised comparative studies (CYA versus BCS and MWA versus BCS), and 22 single-arm trials (eight CYA, six MWA, four RFA, three HIFU and one LA). A list of the trials is available in the Supplementary Material C. Many trials had not reported results despite exceeding their primary completion dates, in some cases, by several years. Additionally, several trials were withdrawn, often citing funding or recruitment challenges, though reasons were often unspecified.

Despite comparative trials of thermal ablation in other tumour entities [[64], [65], [66], [67], [68], [69], [70], [71], [72], [73]], controlled studies in early-stage BC remain limited. This may be attributable to tumour-specific challenges in the breast, including the amorphous and heterogeneous three-dimensional structure of many tumours—particularly infiltrative lobular carcinomas with irregular margins [74]—and the challenges associated with standardising and validating imaging-based assessment of complete tumour destruction [24]. Early thermal ablation studies commonly incorporated surgical excision or biopsy to verify complete ablation histopathologically. However, emerging evidence from prospective non-resection studies suggests that structured MRI- and biopsy-based follow-up may provide a feasible alternative in carefully selected patients [8,38,43,63]. Further standardisation and validation of imaging-based assessment strategies remain necessary. As future randomized trials are likely to compare thermal ablation directly with surgery, rather than combining both interventions, barriers to trial participation may also be reduced. Another methodological challenge is the feasibility of conducting adequately powered RCTs with OS as the primary endpoint. Given the excellent baseline survival in early-stage BC and the expected small effect size of ablation compared with surgery, such trials would require very large sample sizes and prolonged follow-up, posing substantial feasibility challenges. Alternative endpoints — such as DFS, a recognized surrogate for OS in early BC [75,76], or event-free survival [77] —might be prioritised in future trials to improve feasibility. Standardised outcome definitions and consistent reporting of adjuvant therapies (e.g. radiotherapy) would further enhance the interpretability and comparability of future evidence.

Finally, although some patients undergoing thermal ablation may still require axillary staging or treatment, recent trials such as SOUND [78], INSEMA [79], and BOOG 2013-08 [80] support omission of sentinel lymph node biopsy in selected patients with clinically node-negative low-risk early-stage BC. As patients eligible for thermal ablation typically represent a similarly low-risk population, future studies should further evaluate whether axillary surgery can also be safely minimised in this setting.

Additionally, it should be explored which patient groups might benefit most from thermal ablation treatments. Specifically, the suitability of thermal ablation for patients who may not be ideal candidates for traditional surgery due to factors such as age, comorbidities, or personal preferences warrants further investigation.

## 5 Conclusion

Overall, very low to low certainty evidence indicates that three interventions, CYA, MWA and RFA may achieve favourable OS, DFS, recurrence rates, and safety outcomes in selected patients with early-stage BC. However, the available evidence is derived predominantly from small, non-randomised studies with heterogeneous methodologies, varying approaches to complete ablation assessment, and limited long-term comparative follow-up. Further high-quality prospective trials are essential to determine the long-term effectiveness and safety of these interventions compared to traditional surgical methods. Future studies should incorporate clinically relevant oncological endpoints, particularly local recurrence, alongside standardised assessment and follow-up protocols, and evaluate thermal ablation without routine resection to determine the potential of thermal ablation as an alternative to BCS in selected patients with early-stage BC.

## CRediT authorship contribution statement

**Judit Erdos:** Conceptualization, Methodology, Visualization, Writing – original draft. **Julia Kern:** Conceptualization, Project administration, Writing – review & editing. **Doris Giess:** Writing – review & editing. **Andras Drozgyik:** Writing – review & editing. **Gregor Goetz:** Supervision, Writing – review & editing.

## Declaration of competing interest

The authors declare that they have no known competing financial interests or personal relationships that could have appeared to influence the work reported in this paper.
