# Supplementary material for: Thermal ablation for early-stage breast cancer: cryoablation, microwave ablation, radiofrequency ablation, high-intensity focused ultrasound ablation, and laser ablation — a systematic review
Source: Breast. 2026 Jun 16;88:104843. doi: 10.1016/j.breast.2026.104843 (PMC13312544; doi:10.1016/j.breast.2026.104843)
Supplement: Multimedia component 1 [file mmc1.docx]

Supplementary material A

Study extraction tables

Table A - 1: Cryoablation: Results from non-randomised studies of interventions

| **Author, year** | **Galati, 2024 [33]** |
| --- | --- |
| **Country** | Italy |
| **Sponsors** | European Society of Radiology, European Institute for Biomedical Imaging Research, GE Healthcare |
| **Intervention, Product** | CYA, ICEfx Cryoablation System and IceSpehere 1.5 (Boston Scientific, Marlborough, Massachusetts, USA) |
| **Comparator** | Surgery, standard surgery |
| **Study design** | NRSI, prospective, case-control, pilot |
| **Primary study endpoints** | - Presence of necrosis in surgical specimens, - Rate of complete tumour ablation, - Patients’ satisfaction, - Incidence and severity of complications |
| **Guidance, n (%)** | Ultrasound |
| **Resection, n (%)** | **Yes,** within 21 days from enrollment,   - quadrantectomy: 10 (100) vs 9 (90) - MST: 0 (0) vs 1 (10) |
| **(Neo)Adjuvant Therapy, n (%)** | NR |
| **Anaesthesia during ablation, n/N (%)** | Local |
| **Inclusion criteria** | **Intervention:**   - ≥ 18 years - solitary invasive BC (T1 N0) ≤2 cm - ≥ 1.5 cm tumour to skin surface distance and ≥2cm tumour edge to nipple distance - Not eligible for neoadjuvant therapy.   **Control**:   - ≥ 18 years - Early-stage invasive BC (T1 N0) ≤2 cm - Without a cryo-feasible cancer location. |
| **Exclusion criteria** | - Pure DCIS lesions (microcalcifications only on mammogram) - Hx of previous BC - Breast implants - C/I to use of contrast - Non-suitability for cryoablation Tx - Pregnancy or breastfeeding |
| **Recruitment period** | 07.2022 - 01.2023 |
| **Number of patients** | 20 (10 vs 10) |
| **Number of tumours** | 20 (10 vs 10) |
| **Age of patients, mean/median ± SD (range)** | Mean: 65 (47-80) vs 62 (39-84) |
| **Sex, menopausal status** | Female: 10 vs 10,   - Menopausal: 9 (90) vs 6 (60) - Regular menstrual cycle: 1 (10) vs 4 (40) |
| **BC type, n (%)** | **Histology**   - No special type: 8 (80) vs 7 (70) - ILC: 2 (20) vs 1 (10) - Apocrine carcinoma: 0 (0) vs 1 (10) - Mucinous carcinoma: 0 (0) vs 1 (10)   **Tumour grade**   - G1: 1 (10) vs 2 (20) - G2: 8 (80) vs 4 (40) - G3: 1 (10) vs 4 (40) |
| **Size of tumour (mm),**  **mean/median ± SD (range) / n (%)** | Mean: 9.9 (6-18) vs 10.5 (6-13) |
| **Number of sessions in number of patients** | N sessions per patient NR; Procedure time ca. 25 min, 2 freeze cycles (10/5/10 min) |
| **Length of follow-up** | Max. 21 days |
| **Loss to follow-up, n (%)** | 0 |
| **Outcomes** | |
| **Efficacy** | |
| **Mortality** | ≤21 days: 0 vs NR |
| **Complete ablation, n (%)** | ≤21 days**:** 9 (90) vs NA |
| **Determination of complete ablation** | Histologic |
| **Residual tumour, n (%)** | ≤21 days: 1 (10) vs NA |
| **Recurrence, n (%)** | NR |
| **Cosmetic results, n (%)** | **Cosmetic satisfaction** (on a scale of 1-10):   - 8: 5 (50) - 9: 2 (20) - 10: 3 (30) |
| **Quality of life** | NR |
| **Safety** | |
| **Adverse events (overall), n (%)** | Post procedure:  **Minor complications**: 2 (20) vs 0   - Small post-ablative hematoma (about 4 cm in size): 2 (20) vs 0   Pain during cryoablation vs after surgery (1-10)^a^:   - 1: 2 (20) vs 0 - 2: 3 (30) vs 0 - 3: 3 (30) vs 4 (40) - 4: 1 (10) vs 0 - 5: 0 vs 1 (10) - 6: 0 vs 2 (20) - 7: 1 (10) vs 3 (30) - Median: 3 (mild pain) vs 5 (moderate pain)   1 week**:** 0 vs 0  **Pain**:   - 1: 2 (20) vs NR - 2: 8 (80) vs NR |
| **Serious adverse events** | 0 |

*Abbreviations: BC – breast cancer, C/I – contraindication, CYA – cryoablation, DCIS – ductal carcinoma in situ, Hx – history, ILC – invasive lobular carcinoma, MST – mastectomy, n – number, NR – not reported, NRSI – non-randomised study of intervention, SD – standard deviation, Tx – therapy*

*Comments:*

*^a^ Higher scores signify more pain.*

*Table A - 2:* *Cryoablation: Results from single-arm trials (1/4)*

| **Author, year** | **Cazzato, 2015 [34]** | **Poplack, 2015 [41]** | **Simmons, 2016 [43]** |
| --- | --- | --- | --- |
| **Country** | France | USA | USA |
| **Sponsor** | NR | Sanarus Medical, Norris Cotton Cancer Center at the Dartmouth Hitchcock Medical Center (grant No. CA23108) | National Cancer Institute (grant No. U10CA180821 and U10CA180882), Alliance for Clinical Trials in Oncology, Sanarus Technologies |
| **Intervention, Product** | RFA, IceSphere (Galil Medical Ltd., Israel), IceRod (Boston Scientific, Marlborough, Massachusetts, USA) | RFA, Visica (n = 15) or Visica 2 (n=5) treatment system (Sanarus Technologies Inc., Pleasanton, CA). | RFA, Visica 2TM Treatment System (Sanarus Technologies Inc., Pleasanton, CA) |
| **Comparator** | None | None | None |
| **Study design** | Single-arm, prospective, single-centre | Single-arm, prospective, multicentre | Single-arm, non-randomised, multicentre |
| **Primary study endpoints** | Rate of complete tumour ablation | NR | Rate of complete tumour ablation |
| **Guidance, n (%)** | Ultrasound/computed tomography | Ultrasound | NR |
| **Resection, n (%)** | **No** | **Yes,** CE-MRI 25–40 days after ablation, followed within 1–5 days by surgical resection,   - Lumpectomy: 19 (95) - MST: 1 (5) | **Yes**, within 28 days after ablation.   - Partial MST: 85 (98) - Full MST: 2 (2) |
| **(Neo)Adjuvant Therapy, n (%)** | Neoadjuvant: 11 (47)   - endocrine: 11 (47)   Adjuvant: 5 (22)   - endocrine: 5 (22) | - Neoadjuvant: 0 (0) (exclusion criteria) - Adjuvant: NR | - Neoadjuvant: NR - Adjuvant: 86 (100) |
| **Anaesthesia during ablation, n/N (%)** | Local: 18/23 (78.3)  Local + conscious sedation: 5/23 (21.7) | Local | NR (described in protocol, which couldn't be located) |
| **Inclusion criteria** | Unifocal BC ≤3.0 cm   - Tumour to skin surface, nipple and chest wall ≥0.5 cm - Patients declining (1/23) or unsuitable for surgery (19/23) | Unifocal IDC ≤1.5cm (with DCIS component ≤ 25%)   - Tumour to skin surface ≥0.5cm - Enhancement on CE-MRI | Unifocal IDC ≤2.0 cm   - <25% intraductal component - Tumour enhancement visible on MRI |
| **Exclusion criteria** | Multi-focal tumours   - Tumours undetectable with DCE-MRI - Local/systemic infections and/or coagulopathies | Planned neoadjuvant Tx   - Current use of immunosuppressive medications - Breast implants - Angiolymphatic invasion*   * additional exclusion criterion added midway through the study. | Lobular histology: 2   - Lacking tumour enhancement: 1 - Wrong MRI study: 1 - Did not undergo cryoablation: 2 - Surgery instead: 1 - Non credentialed surgeon: 1 - Benign lesion: 1 - Withdrew consent: 1 - Probe failure: 1 - Treatment prior study start: 1 |
| **Recruitment period** | 01.2013 - 01.2015 | NR | 03.2009 - 06.2013 |
| **Number of patients** | 23 | 20 | 86 |
| **Number of tumours** | 23 | 20 | 87*  *one patient had bilateral tumours |
| **Age of patients, mean/median ± SD (range)** | Median: 85 (56-96) | Median: 61 (36-91) | Mean: 61.1±9.3 (42-81)  Median: 62 |
| **Sex, menopausal status** | Female, postmenopausal | Female, NR | Female, NR |
| **BC type, n (%)** | **Histology**   - IDC: 21 (91.3) - ILC: 2 (8.7)   **Tumour grade**   - Grade 1: 7 (30.4) - Grade 2: 10 (43.5) - Grade 3: 4 (17.4) | **Histology**   - IDC: 10 (50) - IDC and ≤25% DCIS: 10 (50) - ER+, PR+, HER2–*: 17 (85) - ER+, PR–, HER2– : 2 (10) - ER+, PR+, HER2+ : 1 (5)   *in the original paper the authors used the alternative name for the HER2 receptor - ERBB2, but we changed it to HER2 to improve understanding and readability | **Histology**   - IDC: 86 (98.9) - other: 1 (1.1)   **Tumour grade**   - G1: 30 (38.0) - G2: 35 (44.3) - G3: 14 (17.7) - unknown: 8   **Receptor status**   - HER2+: 11(12.6) - HER2-, HR+: 75 (86.2) - Triple negative: 1 (1.2) |
| **Size of tumour (mm),**  **mean/median ± SD (range) / n (%)** | Median: 14 (5-28) | Median:   - Mammo: 10 - MRI: 11 - US: 9 | Mean:   - US: 10±4 (0-2) - Mammo: 11±4 (0-19)   Median:   - US: 10 - Mammo: 11 |
| **Number of sessions in number of patients** | Freeze-thaw cycles in patients:   - 2: 10 (43%); (10/10/10) - 3: 13 (57%); (3/3/7/7/7) | 1 session per patient, 2 freeze cycles, time dependent on tumour size and used system:  **Viscia:**   - <10 mm: 8/10/8 min - 10-15 mm: 10/10/10 min   **Viscia 2:**   - <10 mm: 6/10/6 min - 10-15 mm: 8/10/8 min | NR  *Couldn't locate protocol. |
| **Length of follow-up** | 3, 12, 18, 28 months  Median: 14.6 months | 1 day,  7-10 d,  2 weeks.  CE-MRI was performed 25–40 days after ablation, followed within 1–5 days by surgical resection. | Max 28 days |
| **Loss to follow-up, n (%)** | NR | 0 | 0 |
| **Outcomes** | | | |
| **Efficacy** | | | |
| **Mortality** | 2 months: 1 (4)*  *death due to myocardial infarction, unrelated to intervention | <2 months: 0 (0) | ≤28 days: 0 |
| **Complete ablation, n (%)** | NR | <1-2 months: 17 (85) | ≤28 days**:** 80 (92)  including cancer tissue identified >2cm of ablation zone with necrosis of ablated cancer: 66 (76) |
| **Determination of complete ablation** | Imaging | Histologic | Histologic |
| **Residual tumour, n (%)** | NR | <1-2 months: 3 (15) | ≤28 days: 21 (24) |
| **Recurrence, n (%)** | ≤24 months: 5 (22) | NR | NR |
| **Cosmetic results, n/N (%)** | NR | NR | NR |
| **Quality of life** | NR | NR | NR |
| **Safety** | | | |
| **Adverse events (overall), n (%)** | **Immediate** complications: 5 (22)   - Hematomas: 4 (17) - Skin retraction: 1 (4) - Skin burn: 1 (4)   **3 months**: 1 (4)   - Skin retraction: 1 (4) | **1 day**:   - Slight ecchymosis and swelling, no pain: 8 (40) - Minor ecchymosis, pain not requiring analgesics: 4 (20) - Moderate ecchymosis, swelling at cryoablation site, pain requiring over-the-counter analgesics: 8 (40)   **7-10 days**:   - Slight ecchymosis and swelling, no pain: 12 (60) - Minor ecchymosis, pain not requiring analgesics: 6 (30) - Moderate ecchymosis, swelling at cryoablation site, pain requiring over-the-counter analgesics: 2 (10)   **2 weeks:**   - Slight ecchymosis and swelling, no pain: 5 (74) - Minor ecchymosis, pain not requiring analgesics: 4 (21) - Moderate ecchymosis, swelling at cryoablation site, pain requiring over-the-counter analgesics: 1 (5) | NR |
| **Serious adverse events** | 0 | NR | NR |

*Abbreviations: BC – breast cancer, CA – California, CE-MRI – contrast-enhanced magnetic resonance imaging; DCE-MRI – dynamic contrast-enhanced magnetic resonance imaging, DCIS – ductal carcinoma in situ, ER – oestrogen receptor, G1, G2, G3 – tumour grades 1, 2, 3, HER2 – human epidermal growth factor receptor 2, HR – hormone receptor, IDC – invasive ductal carcinoma, ILC – invasive lobular carcinoma, MRI – magnetic resonance imaging, MST – mastectomy, n/N – number of cases / total cases, NR – not reported, PR – progesterone receptor, RFA – radiofrequency ablation, Tx – treatment, US – ultrasound*

Table A - 3: Cryoablation: Results from single-arm trials (2/4)

| **Author, year** | **Fine, 2021 [35]; Fine, 2024 [36]** | **Habrawi, 2021 [37]** | **Kwong, 2023 [40]** |
| --- | --- | --- | --- |
| **Country** | USA | USA | China |
| **Sponsor** | IceCure Medical Ltd. | ASCO Endowment for Excellence in Women’s Health, Sanarus Technologies, Inc. | Li Shu Pui Medical Foundation, the University of Hong Kong Li Ka Shing Faculty of Medicine, the Hong Kong SAR Government (grant No. 0617656), ICeCure Medical Ltd. |
| **Intervention, Product** | RFA, ProSense Cryosurgical System (IceCure Medical Ltd, Caesarea, Israel) | RFA, Visica® 2 Treatment System (Sanarus Technologies Inc., Pleasanton, CA) | RFA, ProSense Cryoablation System (IceCure Medical, Caesarea, Israel) |
| **Comparator** | None | None | None |
| **Study design** | Single-arm, prospective, multicentre, single-arm, non-randomised | Single-arm, prospective, longitudal | Single-arm, prospective, single arm |
| **Primary study endpoints** | Ipsilateral breast tumour recurrence at 5 years, as defined by biopsy | NR | Rate of complete tumour ablation |
| **Guidance, n (%)** | Ultrasound | Ultrasound | Ultrasound |
| **Resection, n (%)** | **No** | **No** | **Yes**, 8 weeks after ablation,   - lumpectomy: 15 (8) |
| **(Neo)Adjuvant Therapy, n (%)** | Neoadjuvant**:** 0 (0) (exclusion criteria)  Adjuvant: 153 (79)   - Endocrine only: 124 (64) - Whole-breast radiation only: 3 (2) - Endocrine + radiation: 25 (13) - Endocrine + radiation + chemo: 1 (1) | Neoadjuvant**:** NR  Adjuvant**:** 12 (100)   - radiation: 1 (8) - endocrine: 12 (100) | NR |
| **Anaesthesia during ablation, n/N (%)** | NR | NR | General |
| **Inclusion criteria** | - Female, ≥60 years - Unifocal IDC ≤1.5 cm - ER+, PR+, HER2- - Low to intermediate histology grade (B2) - Clinically confirmed negative axillary status (US and palpation) | - Patients ≥50 years - Unifocal IDC ≤1.5 cm (without extensive in situ component visible on US) - ER+, PR+, HER2- | - Solitary T1 BC - Tumour to skin surface distance ≥0.5 cm - Any immunohistotype |
| **Exclusion criteria** | - Multifocal and/or multicentric tumours - ≥25% intraductal component - Prior surgical biopsy - Neoadjuvant Tx - Coagulopathy or thrombocytopenia - Non-suitability for cryoablation Tx | NR | - Invasive lobular carcinoma - Lobular carcinoma in situ - Retro areolar tumour - Pregnancy or breastfeeding |
| **Recruitment period** | 10.2014 - 02.2019 | 01.2017 - 02.2020 | 2018 - NR |
| **Number of patients** | 194 | 12 | 15 |
| **Number of tumours** | 194 | 12 | 15 |
| **Age of patients, mean/median ± SD (range)** | Mean: 74.9±6.9 (55-94) | Mean: 74.1±10.3 (55-93)  Median: 75 | Median: 53 (40-67) |
| **Sex, menopausal status** | Female, NR | Female, NR | NR, NR |
| **BC type, n (%)** | **Tumour grade**   - Grade 1: 98 (51) - Grade 2: 96 (49)   **Receptor status**   - ER+: 194 (100) - PR+: 184 (92.8) - HER2–: 194 (100) | **TNM Staging**   - Stage 1A: 7 (58.3) - Stage 2B: 4 (33.3) | **Histology**   - DCIS: 5 (33.3) - IDC: 10 (66.6)   **Receptor status***   - HR+, HER2-: 3 (30) - HR+, HER2+: 2 (20) - HER2 enriched: 3 (30) - Triple negative: 2 (20)   *receptor status only reported on 10 IDC patients |
| **Size of tumour (mm),**  **mean/median ± SD (range) / n (%)** | Mean:  US:   - Sagittal: 8.0±2.9 (2.5-14.9) - Transverse: 7.4±2.7 (2.8–14) - Anterior-posterior: 6.3±2.6 (1-14) - median:  US: - Sagittal: 8.1 - Transverse: 7.0 - Anterior-Posterior: 6.3 | Mean: 9.9±2.9 (5-15)  Median: 10 | Median:   - MRI: 16 (10-20) - US: 13 (8.6-18) |
| **Number of sessions in number of patients** | 1 session per patient, 2 freeze cycles, (9/8/9) | 1 session per patient, freeze cycles depending on tumour size:   - <10 mm: 6/10/6 min - 10-20 mm: 8/10/8 min | Median procedure time: 75 (25-101) min, freeze-thaw cycles in patients:   - 2: 10 (67) - ≥3: 5 (33) |
| **Length of follow-up** | 6, 12, 24, 36, 48, 60 months   - Interim: mean: 34.8±18 months - Mean: 54±13.07 months | 24-48 hours, 2 weeks, 6, 12, 18, 24 months | 6 weeks (MRI scans), 8 weeks (lumpectomy procedure) |
| **Loss to follow-up, n (%)** | 3 years (interim): 18 (9)  5 years: 32 (16) | 6 months: 1 (8)  12 months: 4 (33)  24 months: 8 (67) | 0 |
| **Outcomes** | | | |
| **Efficacy** | | | |
| **Mortality** | 3 years:   - Unrelated to BC*: 10 (5)   * Reasons unrelated to the device, procedure, or BC  5 years: 21 (11)   - Distant metastasis: 2 (1) - Unknown reasons: 3 (2) - Unrelated to BC*: 16 (8)   *due to heart failure, respiratory failure, myocardial infarction, cardiac arrest, non-traumatic intracerebral hemorrhage, and renal failure leading to multiorgan failure  BC survival rate: 96.7 (95% CI 92.2-98.6%)  Overall survival rate: 88.6% (82.9-92.5%) | 6 months: 0 (0)  12 months: 0 (0)  24 months**:** 0 (0) | 8 weeks: 0 (0) |
| **Complete ablation, n (%)** | NR | 6 months: 11 /11 (100)  12 months: 8/8 (100)  24 months: 4/4 (100) | 8 weeks: 8 (53) |
| **Determination of complete ablation** | NA | Imaging (mammography+US+MRI)*  *only if findings suspicious, core needle biopsy: 4 patients had histopathological evaluation by biopsy | Histologic |
| **Residual tumour, n (%)** | NR | 6 months: 0 (0)  12 months: 0 (0)  24 months: 0 (0) | 8 weeks: 7 (47) |
| **Recurrence, n (%)** | Mean FU: 34.83±17.96 months: (0.07–67.55):   - local recurrence: 4 (2.06, 95% CI, 0.56–5.19)   36 months:   - local recurrence: 1 (0.6, 95% CI 0.1–3.9)   48 months:   - local recurrence: 3 (1.7, 95% CI 0.6–5.3)   Mean FU: 54.16±13.07 months:   - local recurrence: 7 (3.61)   5 years (60 months):   - local recurrence: 8 (4.3, 95% CI 2.1–8.7) | 6 months: 0 (0)  12 months: 0 (0)  24 months: 0 (0) | NR |
| **Cosmetic results, n/N (%)** | **Cosmetic satisfaction** (on a scale of 1-5, but given as % of follow-up patients/physicians who were satisfied with the results)  6 months:   - Patients (n=177/194): 99.3% - Physicians (n=176/194*): 98,6%   *patients/procedures  3 years:   - Patients (n=NR): 95% - Physicians (n=NR): 98%   5 years:   - Patients (n=111/194): 100% - Physicians (n=102/194): 100% | No cosmetic deficits in any patient | NR |
| **Quality of life** | NR | NR | NR |
| **Safety** | | | |
| **Adverse events (overall), n (%)** | **3 years** (interim): 43 AEs in 23 (12) patients   - Mild: 38 - Moderate: 5 - Severe: 0   **5 years:**187 AEs in 97 (50) patients  **Mild**: 165   - Bruising: 48 (25.7) - Pain: 39 (20.9) - Oedema: 36 (19.3) - Hematoma: 8 (4.3) - Tenderness: 8 (4.3) - Pruritus and rash: 4 (2.1) - Erythema multiforme: 3 (1.6) - Injection site reaction: 3 (1.6) - Burn: 3 (1.6) - Fatigue: 2 (1.1) - Drainage: 2 (1.1) - Flushing: 1 (0.5) - Skin infection: 1 (0.5) - Breast twitches: 1 (0.5) - Heat sensation: 1 (0.5) - Breast warm to the touch: 1 (0.5) - Tethering: 1 (0.5) - Dimpling: 1 (0.5) - Hemorrhage: 1 (0.5) - Induration at cryo site: 1 (0.5)   **Moderate:** 18   - Bruising: 10 (5.3) - Edema: 3 (1.6) - Burn: 2 (1.1) - Pain: 2 (1.1) - Hematoma: 1 (0.5)   **Severe:** 4   - Bruising: 4 (2.1) | **Post procedure**:  Minor complications: 10 (83)   - Bruising: 5 (42) - Oedema: 2 (16) - Mild to moderate pain: 3 (12)   **2 weeks**: 0 | 0 |
| **Serious adverse events** | 0 | 0 | NR |

*Abbreviations: AE – adverse event, BC – breast cancer, CI – confidence interval, ER – oestrogen receptor, fu – follow-up, HER2 – human epidermal growth factor receptor 2, IDC – invasive ductal carcinoma, MRI – magnetic resonance imaging, n – number, NA – not applicable, NR – not reported, PR – progesterone receptor, RFA – radiofrequency ablation, SD – standard deviation, US – ultrasound, USA – United States of America*

Table A - 4: Cryoablation: Results from single-arm trials (3/4)

| **Author, year** | **Khan, 2023 [39]** | **RocaNavarro, 2024 [42]** | **Kawamoto, 2024 [38]** |
| --- | --- | --- | --- |
| **Country** | USA | Spain | Japan |
| **Sponsor** | ASCO Equipment Endowment for Excellence in Women’s Health, Sanarus technologies Inc. and ICECure Medical (donation of probes) | La Paz University Hospital | None |
| **Intervention, Product** | RFA, Visica 2 treatment system (Sanarus Technologies, Inc. Pleasanton, CA, USA, now acquired by ICECure Medical, Caesarea, Israel) | RFA, ICEfx Cryoablation System (Boston Scientific, Marlborough, Massachusetts, USA), Needles: IceSphere 17G (n=40) or IcePearl 14G (n=20) | RFA, ProSense Cryosurgical System (IceCure Medical Ltd, Caesarea, Israel) |
| **Comparator** | None | None | None |
| **Study design** | Single-arm, prospective, single centre, longitudinal | Single-arm, prospective, observational | Single-arm, single centre |
| **Primary study endpoints** | NR | Presence of residual invasive cancer | Cryolesion due to ablation procedure |
| **Guidance, n (%)** | Ultrasound | Ultrasound | Ultrasound |
| **Resection, n (%)** | **No** | **Yes**, after mean 21.8±13.8 (6-78) days after ablation, BCS: 60 (100) | **No** |
| **(Neo)Adjuvant Therapy, n (%)** | Neoadjuvant**:** NR  Adjuvant**:**   - Endocrine: 31 (97) - Radiation: 6 (19) | NR | Neoadjuvant: NR  Adjuvant**:** 18 (100)   - Radiation: 18 (100) - Endocrine: 18 (100) |
| **Anaesthesia during ablation, n/N (%)** | Local | Local | Local |
| **Inclusion criteria** | - ≥50 years - Unifocal IDC ≤1.5 cm (without extensive in situ components) - ER+, PR+, HER2- | - ≥18 years - IDC ≤2 cm - ER+, HER2- - Radiologically confirmed negative axillary status (US) - Suitable for BCS, with no requirement for primary systemic therapy | - Female, 20-85 years - Unifocal IDC ≤1.5 cm - ER+, PR+, HER2-, ≤20% Ki67+ - Negative SLN biopsy - ECOG performance status 0/1 |
| **Exclusion criteria** | NR | - Tumours ≥2.0 cm with extensive intraductal component - HER2 + luminal tumours - Axillary involvement - Distant metastasis - Pregnancy or breastfeeding | - Invasive lobular carcinoma - Invasive microcapillary carcinoma - Intraductal lesions - Tumour to skin surface and pectoralis <0.5 cm |
| **Recruitment period** | 01.2017 - 05.2023 | 03.2021 - 06.2023 | NR |
| **Number of patients** | 32 | 59 | 18 |
| **Number of tumours** | 33*  *one patient had bilateral tumours | 60*  *one patient had 2 tumours ablated in the same breast | 18 |
| **Age of patients, mean/median ± SD (range)** | Mean: 71±10.5 (50-91)  Median: 70 | Mean: 63±8 (31-81) | Mean: 59±9.0 (43-72)  Median: 60.3 |
| **Sex, menopausal status** | Female, NR | Female, NR | Female, NR |
| **BC type, n (%)** | **TNM Staging**   - Stage T1: 34 (100) - 1A: 9 (27.3) - 1B: 13 (39.4) - 1C: 11 (33.3)   **Tumour grade**   - Grade 1/2: 34 (100) | **Tumour grade**   - G1: 23 (38) - G2: 37 (62)   **Receptor status**   - ER+: 60 (100) - PR+: 53 (88) - HER2-: 60 (100) | **Histology**   - IDC: 17 (94.4) - Mucinous carcinoma: 1 (5.6)   **Receptor status**   - ER+: 18 (100) - PR+: 17 (94.4) - HER2-: 18 (100) |
| **Size of tumour (mm),**  **mean/median ± SD (range) / n (%)** | Mean:   - US: 8.7±3.5 (4.0-15)   Median:   - US: 8.0 | Mean:   - US: 10.1±3.6 (4-20) | Mean:   - MRI: 9.8±2.3 (6-14.5)   Median:   - MRI: 9.9 |
| **Number of sessions in number of patients** | NR | N session per patient NR, 2 freeze-cycles: 10/10/10 min | N session per patient NR |
| **Length of follow-up** | 6, 12, 18, 24 months   - <6 months: 3 (9.4) - 6-12 months: 2 (6.3) - 12-24 months: 8 (25.0) - ≥24 months: 20 (62.5) | Mean: 21.8±13.8 (6-78) days | 1, 6, 12, 24, 36, 60 months   - mean: 34.5±16.2 (18-68) - median: 44.3 |
| **Loss to follow-up, n (%)** | NR | 0 | 0 |
| **Outcomes** | | | |
| **Efficacy** | | | |
| **Mortality** | 1 year**:** 1 (3)*  *death due to unrelated causes | NR | Mean FU 34.5 months: 0 |
| **Complete ablation, n (%)** | NR | 55 (92) | NR |
| **Determination of complete ablation** | NA | Histologic | Imaging |
| **Residual tumour, n (%)** | NR | Mean FU: 5/60 | 0 (0) |
| **Recurrence, n (%)** | 18 months:   - Local recurrence: 0 (0) - Distant metastasis: 1 (3) | NR | 5 years:   - Local recurrence: 0 (0) - Distant metastasis: 0 (0) |
| **Cosmetic results, n/N (%)** | NR | NR | Visual assessment (by Moire topography):  excellent results with no nipple positions distortion, breast deformity or asymmetry observed (no numerical results) |
| **Quality of life** | NR | NR | **EQ-VAS** (mean baseline: 84)   - 36m: 85 - 60m: 82.5   **EQ-5D-5L** (mean baseline: 0.9):   - 6m: 0.89 - 12m: 0.87 - 24m: 0.94 - 36m: 0.94 - 60m: 0.93 |
| **Safety** | | | |
| **Adverse events (overall), n (%)** | NR | **Post procedure:**  *Mild AEs:* 4 (7)*   - Mild discomfort: 6 (10) - Moderate to severe pain: 1 (2) - Small (5 mm) skin vesicle: 1 (2)   *authors did not define what the mild AEs were and reported 8 AEs in text | **1 week:**  *Overall***:** 1 (5.5)   - Skin redness grade 1 (CTCAE classification): 1 (5.5)   *varying degrees of burns in the pectoralis muscle were observed by MRI 1 month post intervention in all patients but were symptomless and resolved after 6 months |
| **Serious adverse events** | NR | NR | 0 |

*Abbreviations: AE – adverse event, ASCO – American Society of Clinical Oncology, BC – breast cancer, BCS – breast-conserving surgery, CTCAE – common terminology criteria for adverse events, ECOG – Eastern Cooperative Oncology Group, ER – oestrogen receptor, EQ-5D-5L – euroqol 5-dimension 5-level questionnaire, EQ-VAS – euroqol visual analogue scale, FU – follow-up, G1, G2, G3 – tumour grades 1, 2, 3, HER2 – human epidermal growth factor receptor 2, IDC – invasive ductal carcinoma, MRI – magnetic resonance imaging, NA – not applicable, NR – not reported, PR – progesterone receptor, RFA – radiofrequency ablation, SD – standard deviation, SLN – sentinel lymph node, TNM – tumour-node-metastasis staging system, Tx – treatment, US – ultrasound*

*Table A - 5: Cryoablation: Results from single-arm trials (4/4)*

| **Author, year** | **Holmes, 2026 [44] FROST trial (NCT01992250)** | **Wooldrik, 2025a [46], 2025b [45], THERMAC trial (Dutch trial registry NL9205)** |
| --- | --- | --- |
| **Country** | USA | The Netherlands |
| **Sponsor** | Sanarus Technologies, Inc. | Stichting Borstkankeronderzoek Rotterdam, Stichting Team Westland, Stichting Bevordering Onderzoek Franciscus, Stichting Maurits en Anna de Kock, Stichting Vrienden van het Havenziekenhuis, and Stichting Coolsingel.  IceCure Medical loaned equipment for the research. |
| **Intervention, Product** | Cryoablation (CYA) using Visica2 Cryoablation System (Sanarus Medical, Inc., Pleasanton, CA, USA), using liquid nitrogen and a single 10-gauge cryoprobe | Cryoablation using ProSense generator (IceCure Medical) with 10-gauge cryoprobe |
| **Comparator** | None | None |
| **Study design** | Prospective, phase 2, multicentre, non-randomized study,  with participants stratified into two age-based cohorts, study arm 1 (low risk) and 2 (higher risk). | Prospective, open-label, multicentre, phase 2, randomized study*  *study comparing CYA, MWA and RFA. In our scope we treat this study as single-arm for these interventions separately. |
| **Primary study endpoints** | Rate of successful tumour ablation (i.e., response rate) at 6 months, defined as the absence of residual viable invasive or in situ carcinoma detected by core biopsy of the cryoablation zone 6 months after cryoablation | Rate of pathologic complete response (pCR), defined as the absence of viable tumour assessed with cytokeratin 8 and 18 (CK8/18) and hematoxylineosin staining. |
| **Guidance, n (%)** | US | US |
| **Resection, n (%)** | **No** | **Yes**, BCS or MST 3 months after the thermal ablation |
| **(Neo)Adjuvant Therapy, n (%)** | Study arm 1: 5-year course of endocrine therapy  Study arm 2: radiotherapy + 5-year course of endocrine therapy | NR |
| **Anaesthesia during ablation,** | Local | Local |
| **Inclusion criteria** | - ≥50 years - Unifocal clinical stage I (T1, ≤2.0 cm), - ER+, PR+, HER2- IDC confirmed by core biopsy - no evidence of axillary adenopathy by physical examination and axillary ultrasound | - Postmenopausal women - Unilateral luminal cT1N0M0, - US visible/MRI confirmed tumour ≤2.0 cm |
| **Exclusion criteria** | - Prior treatment (e.g., open surgical biopsy, lumpectomy) of index cancer - Ductal carcinoma in-situ with microinvasions (T1mic) - Multifocal or multicentric invasive breast carcinoma - Prior or planned neoadjuvant systemic therapy for breast cancer - Component of DCIS more than 25% of the tumour area | - Additional malignancies confirmed by MRI (e.g., satellite lesion or contralateral tumour), - Component of DCIS more than 25% of the tumour area - Features incompatible with thermal ablation such as invasion in the skin or areola that were not apparent at US or mammography. |
| **Recruitment period** | 07.2016 – 10.2020 | 03.2021 - 05.2024 |
| **Number of patients** | 85*  *analyses carried out on the patients with complete follow-up (83) | 18 |
| **Number of tumours** | 85 | 18 |
| **Age of patients, mean/median ± SD (range)** | Study arm 1: Mean 76 ± 4, Median 76 (70–95)  Study arm 2: Mean 62 ± 5, Median 62 (51–69)  Total: Mean 70 ± 9, Median 71 (51–95) | Mean 67.9 ± 10.5 |
| **Sex, menopausal status** | Female, NR | Female, postmenopausal |
| **BC type, n (%)** | **Histology**   - Infiltrating ductal carcinoma: 83 (100)   **Grade**   - Low: 34 (41.1) - Intermediate: 47 (56.8) - High: 2 (2.1)   **Hormone receptor status**   - ER+/PR+: 80 (96.5) - ER+/PR–: 3 (3.6) - ER–/PR+: 0 (0) - HER2–: 83 (100) | **Grade**   - 1: 7 (39) - 2: 11 (61)   **Hormone receptor status**   - ER+/PR+/HER2−: 16 (89) - ER+/PR−/HER2−: 2 (11) |
| **Size of tumour (mm),**  **mean/median ± SD (range) / n (%)** | US:  Median: 9.0 (4–19)  Mean: 9.3 ± 3.2 | US:  Mean: 9.7 ± 4.2 (4–16) |
| **Number of sessions in number of patients** | Session per patient NR, 2 freeze-cycles (freezing/thawing/freezing): 10/10/10 min | Session per patient NR, 2 freeze-cycles (freezing/thawing/freezing): 9/8/9 min |
| **Length of follow-up** | Median 6.11 years for the overall study population  (5.91 years for study arm 1 and 6.38 years for study arm 2) | 3 months |
| **Loss to follow-up, n (%)** | 2 | 0 |
| **Outcomes** | | |
| **Efficacy** | | |
| **Mortality, n (%)** | Median FU of 6.11 years: 3 (4) *  *death due to unrelated causes  5-year OS Kaplan–Meier estimate: 96.4% for the combined cohorts, 97.92% for study arm 1, and 94.20% for study arm 2. | 3 months: 0 (0) |
| **Complete ablation, n (%)** | **6 months**:  Study arm 1: 47 (98)*  Study arm 2: 35 (100)  Total: 82/83 (99)  *1 patient declined core needle biopsy, which would confirm complete ablation. | **3 months**:  17 (94) |
| **Determination of complete ablation** | Histopathologic: Core needle biopsy 6 months after ablation. | Histopathologic examination 3 months after ablation (viable tumour assessed with cytokeratin 8 and 18 and hematoxylineosin staining) |
| **Residual tumour, n (%)** | 6 months: 0/82 (0)*  *1 patient did not go under core needle biopsy. | 1 (6)*  *1 patient underwent early surgery |
| **Recurrence, n (%)** | 5 years:  IBTR recurrence (≤5 from the original tumour site): 3 (4)  IBTR recurrence (>5 cm from the original tumour site): 1 (1)  Ipsilateral axillary recurrence: 2 (2)  5-year IBTR recurrence Kaplan–Meier estimate: 3.6% (95% CI, 0% to 7.6%) | NR |
| **Cosmetic results, n/N (%)** | NR | Overall cosmetic outcomes were rated as good after thermal ablation (not reported per study arm) and intermediate after surgery (BCTOS median 1.6 vs 1.8). BCCT.core rated 94% of cases as good/excellent after thermal ablation versus 80% after surgery. |
| **Quality of life** | NR | BREAST-Q psychosocial, sexual well-being and satisfaction domains were generally higher after thermal ablation (not reported per study arm) than after surgery. |
| **Safety** | | |
| **Adverse events (overall), n (%)** | Cryoablation-related AEs^[[1]](#footnote-1)^  **2 weeks:**  *Grade 1*:   - breast pain: 26 (32) - bruising/hematoma: 18 (22) - wound infection: 4 (5) - localized oedema: 6 (7) - postoperative haemorrhage: 2 (3) - skin ulceration: 2 (3) - dizziness: 2 (3)   *Grade 2*:   - breast pain: 5 (7) - wound infection: 1 (2) - bruising/hematoma: 1 (2) - localized oedema: 2 (2)   *Grade 3*: 0 (0)  **6 months:**  *Grade 1*:   - breast pain: 11 (13) - bruising/hematoma: 7 (8) - localized oedema: 7 (8) - nausea: 4 (5)   *Grade 2*:   - breast pain: 9 (11) - dizziness: 7 (8) - headache: 4 (5)   *Grade 3:* 0 (0) | 0 (0) |
| **Serious adverse events** | NR | 0 (0) |

*Abbreviations: AE – adverse event, BCCT.core - Breast Cancer Conservative Treatment. cosmetic results, BCS – breast-conserving surgery, BCTOS - Breast Cancer Treatment Outcome Scale, CI – confidence interval, CYA – cryoablation, DCIS - ductal carcinoma in situ, ER – oestrogen receptor, FU – follow-up, HER2- – human epidermal growth factor receptor 2 negative, IBTR - ipsilateral breast tumour recurrence, IDC – invasive ductal carcinoma, MWA – microwave ablation, MRI – magnetic resonance imaging, MST – mastectomy, NR – not reported, OS – overall survival, pCR - pathologic complete response, PR – progesterone receptor, RFA – radiofrequency ablation, SD – standard deviation, US – ultrasound*

Table A - 6: Microwave ablation: Results from non-randomised studies of interventions

| **Author, year** | **Zhong, 2023 [47]** |
| --- | --- |
| **Country** | China |
| **Sponsor** | National Natural Science Foundation of China (grant No. 81771953 and 82172683), the Natural Science Foundation of Jiangsu Province (grant No. BK20180108), the Priority Academic Program Development of Jiangsu Higher Education Institutions |
| **Intervention, Product** | MWA, Nanjing Yigao Microwave Electric Institute, China |
| **Comparator** | Surgery, MST or BCS and axillary lymph node dissection or SLNB with adjuvant therapy |
| **Study design** | Propensity score matched NRSI, prospective, non-randomised, multicentre controlled |
| **Primary study endpoints** | DFS, OS, LOS |
| **Guidance, n (%)** | Ultrasound |
| **Resection, n (%)** | **No** |
| **(Neo)Adjuvant Therapy, n (%)** | Neoadjuvant: 0 (0) (exclusion criteria)  Adjuvant: 33 (100) vs 99 (100)   - Endocrine: 33 (100) vs 99 (100) - Radiation: 0 (0) vs 8 (8), p=0.20 - Chemo: 0 (0) vs 8 (8), p=0.20 |
| **Anaesthesia during ablation, n/N (%)** | Local |
| **Inclusion criteria** | - Female, >70 years - Single invasive BC ≤3.0 cm - HR+/- - Clinically negative axillary LN (US) - Tumour outside the nipple/areola area with any distance to the skin and chest wall but not infiltrated to skin and pectoralis muscle |
| **Exclusion criteria** | - Previous surgery, - Radiotherapy or systemic antitumour Tx - Distant metastasis |
| **Recruitment period** | 01.2016 - 07.2021 |
| **Number of patients** | 132 (33 vs 99) |
| **Number of tumours** | 132 (33 vs 99) |
| **Age of patients, mean/median ± SD (range)** | Mean: 77.9±7.6 (70-94) vs 77.4±5.5 (70-92) |
| **Sex, menopausal status** | Female, postmenopausal |
| **BC type, n (%)** | **Histology***,* p=0.147   - IDC: 31 (94) vs 80 (81) - ILC: 1 (3) vs 4 (4) - other: 1 (3) vs 15 (15)   **ER***,* p=1.000   - <90%: 2 (6.1) vs 8 (8.1) - ≥90%: 31 (93.9) vs 91 (91.9)   **PR*,*** p=0.619   - <20%: 8 (24.2) vs 19 (19.2) - ≥20%: 25 (75.8) vs 80 (80.8)   **Ki67 status***,* p=0.681   - ≤14%: 13 (39.4) vs 35 (35.4) - >14%: 20 (60.6) vs 64 (64.6) |
| **Size of tumour (mm),**  **mean/median ± SD (range) / n (%)** | <1cm: 2 vs 7,  1-2cm: 18 vs 5,  >2cm: 13 vs 35, p=0.948  **Intervention group** (mm):   - mean: 18.9±5.9 (7-30) - median: 18 |
| **Number of sessions in number of patients** | Sessions per patients:   - 1: 32 (97) - 2: 1 (3)*   *1 week after first ablation, due to residual tumour on US  mean ablation time: 2.64±0.59 (1.67-4.5) min |
| **Length of follow-up** | Median: 31 (2-74) months |
| **Loss to follow-up, n (%)** | 0 |
| **Outcomes** | |
| **Efficacy** | |
| **Mortality** | OS median FU:   - MWA: 0/33 died - Control: 1/99 died   HR: 0.537, 95% CI: 0.089-3.325, p=0.49  OS rate 1 year:   - MWA: 97% - Control: 100%   OS rate 3 year:   - MWA: 92.6% - Control: 96.1% |
| **Complete ablation, n (%)** | 1 week: 32 (97)  1 month: 33 (100) (95% CI, 89.4-100%) |
| **Determination of complete ablation** | Imaging |
| **Residual tumour, n (%)** | 1 week: 1 (3)* vs NR  1 month: 0 vs NR  *The patient received second ablation. |
| **Recurrence, n (%)** | **Overall tumour progression,** median FU: 1 (3) vs 3 (3)  HR: 0.536; 95%, CI 0.128-2.249, p=0.38   - Local recurrence: 1 (3) vs 1 (1) - Distant metastasis: 0 (0) vs 2 (2) |
| **Cosmetic results, n/N (%)** | NR |
| **Quality of life** | NR |
| **Safety** | |
| **Adverse events (overall), n (%)** | 0 vs NR |
| **Serious adverse events** | 0 vs NR |

*Abbreviations: AE – adverse event, BC – breast cancer, BCS – breast-conserving surgery, CI – confidence interval, DFS – disease-free survival, ER – oestrogen receptor, FU – follow-up, HR – hazard ratio, HR+/- – hormone receptor positive/negative, IDC – invasive ductal carcinoma, ILC – invasive lobular carcinoma, Ki67 – proliferation marker Ki-67, LN – lymph node, LOS – length of stay, MWA – microwave ablation, MST – mastectomy, NRSI – non-randomized study of interventions, NR – not reported, OS – overall survival, PR – progesterone receptor, SD – standard deviation, SLNB – sentinel lymph node biopsy, Tx – treatment, US – ultrasound*

Table A - 7: Microwave ablation: Results from single-arm trials

| **Author, year** | **Ji, 2024 [48]** | **Zhou, 2021 [50]** | **Pan, 2024 [49]** | **Wooldrik, 2025a [46], 2025b [45], THERMAC trial (Dutch trial registry NL9205)** |
| --- | --- | --- | --- | --- |
| **Country** | China | China | China | The Netherlands |
| **Sponsor** | National Natural Science Foundation of China (grant No.12090024, 81972872), Science and Technology Innovation Project of Shanghai Science and Technology Commission (grant No.17441900700) | National Natural Science Foundation of China (grant No. 81771953), the Six Kinds of Outstanding Talent Foundation of Jiangsu Province (grant No. WSW-014), the Natural Science Foundation of Jiangsu Province (grant No. BK20180108), the Priority Academic Program Development of Jiangsu higher Education Institutions | Natural Science Foundation of Jiangsu Province, the Jiangsu Province Capability Improvement Project, the Jiangsu Province Excellent Postdoctoral Program, Jiangsu Provincial Science and Technology Department, Jiangsu Provincial Science and Technology Department, Jiangsu Postgraduate Practice and Innovation Plan, Priority Academic Program Development of Jiangsu HIgher Education Institutions | Stichting Borstkankeronderzoek Rotterdam, Stichting Team Westland, Stichting Bevordering Onderzoek Franciscus, Stichting Maurits en Anna de Kock, Stichting Vrienden van het Havenziekenhuis, and Stichting Coolsingel.  IceCure Medical loaned equipment for the research. |
| **Intervention, Product** | MWA, Microwave probe: Vision China Medical Devices R&Dm, Najing, China; MR-guided PMC: DynaCAD Version 2.0 (Invivo Corporation, FL, USA) | MWA, Nanjing Yigao Microwave Electric Institute, China | MWA, Nanjing Yigao Microwave Electric Institute, China | AMICA generator (AGN-H-1.0; HS Hospital Service) with corresponding 16G probes |
| **Comparator** | None | None | None | None |
| **Study design** | Single-arm, prospective, single centre, observational | Single-arm, prospective, observational, single arm, multicentre | Single-arm, RCT, single centre, open label, three arms - cohort | Open-label, phase 2, randomised study*  *study comparing CYA, MWA and RFA. In our scope we treat this study as single-arm for these interventions separately. |
| **Primary study endpoints** | Treatment efficacy | Rate of complete tumour ablation,  (secondary: MWA-induced immune response 1 week after ablation) | Treatment feasibility and safety (secondary: complete ablation rate) | Rate of pathologic complete response (pCR), defined as the absence of viable tumour assessed with cytokeratin 8 and 18 (CK8/18) and hematoxylineosin staining. |
| **Guidance, n (%)** | MRI | US | US*  *Based on information that they followed protocol of their centre (Zhong, 2023) | US |
| **Resection, n (%)** | **Yes**, immediately after ablation,  MST: 26 (100) | **Yes**, 1 week after ablation,   - surgery: 20 (57)   declined surgery/unsuitable for surgery after ablation: 15 (43) | **Yes**, 7-10 days after ablation,   - MST: 16 (40) - BCS: 23 (57.5) | **Yes**, BCS or MST 3 months after the thermal ablation*  *based on information of the study protocol (van de Voort, 2021) |
| **(Neo)Adjuvant Therapy, n (%)** | NR | Neoadjuvant: NR  Adjuvant:  15 patients who underwent MWA without surgery received:   - Endocrine: 14 (93) - Anti-HER2 treatment: 1 (7)   20 patients with MWA + surgery: NR | Neoadjuvant: 1 (5)  Adjuvant: NR | NR |
| **Anaesthesia during ablation, n/N (%)** | Local | Local | Local   *based on information that they followed the protocol of their centre (Zhong, 2023) | Local |
| **Inclusion criteria** | - Unifocal BC ≤2.0 cm - Tumour to skin surface and pectoralis ≥1.0 cm - Radiologically confirmed negative axillary status (US) and free from distant metastasis (CT staging) | - Solitary invasive BC ≤3.0 cm (without extensive intraductal component) - No infiltration of skin or pectoralis muscle | - ≥18 years - Invasive solitary BC ≤3.0 cm - No infiltration of the skin and pectoralis muscle - No systemic therapy - Adequate organ and bone marrow function | - Postmenopausal women with unilateral luminal cT1N0M0 breast cancer, - MRI confirmed tumour 2 cm or smaller |
| **Exclusion criteria** | - Multifocal lesions - Preference to undergo BCS - Pregnancy or breastfeeding | NR | - Inflammatory BC - Prior radiotherapy or use of other - Immunosuppressive agents - Hx of autoimmune disease - Recent vaccination | - Additional malignancies confirmed by MRI (e.g., satellite lesion or contralateral tumour), - Component of ductal carcinoma in situ (DCIS) more than 25% of the tumour area or - Features incompatible with thermal ablation such as invasion in the skin or areola that were not apparent at ultrasound or mammography. |
| **Recruitment period** | 05.2018 - 12.2019 | 07.2016 - 06.2019 | 03.2021 - 08.2022 | 03.2021 – 05.2024 |
| **Number of patients** | 26 | 35 | 40 (20, 20) | 18 |
| **Number of tumours** | 26 | 35 | 40 (20, 20) | 18 |
| **Age of patients, mean/median ± SD (range)** | Mean: 52.0±12.2 (31–75) | Median: 59 (38-87) | Mean:  **MWA:** 52.6 (37-65)  **MWA+cam**: 50.5 (32-64) | Mean: 65.8 ± 8.1 |
| **Sex, menopausal status** | Female, NR | Female, NR | Female, NR | Female, postmenopausal |
| **BC type, n (%)** | **Histology**   - IDC: 20 (77) - Invasive lobular carcinoma: 1 (3.8) - Solid papillary carcinoma: 1 (3.8) - Mucinous carcinoma: 2 (7.7) - DCIS: 2 (7.7)   **Receptor status**   - ER+, PR+/HER2-: 12 (46.2) - ER+, HER2- : 6 (23.1) - ER+, HER2+: 5 (19.2) - HER2 enriched: 1 (3.8) - Triple negative: 2 (7.7) | **Receptor status**   - HR+, HER2-: 20 (57) - HER2+: 7 (20) - Triple negative: 8 (23)   **TNM staging**   - T1: 20 (57) - T2: 15 (43) - N0: 29 (83) - N1: 4 (11) - N2: 2 (6) | **Receptor status**   - HR+, HER2-: 25 (62.5) - HER2+: 8 (20) - Triple negative: 7 (17.5) | **Grade**   - 1: 9 (50) - 2: 9 (50)   **Hormone receptor status**   - ER+/PR+/HER2−: 16 (89) - ER+/PR−/HER2−: 2 (11) |
| **Size of tumour (mm),**  **mean/median ± SD (range) / n (%)** | Mean: MRI:  Long axis: 14.88±2.55 (11–19)  Short axis: 12.19±3.25 (5–19) | **MWA:** 18.8  **MWA+surgery:** 19.6  T1: 20 (57.1)  T2: 15 (42.9) | US ≤20 mm: 29 (72.5)  >20 mm: 11 (27.5) | US:  11.4 ± 4.4 (4–20) |
| **Number of sessions in number of patients** | 1 session per patient,  Mean ablation time: 4.2±0.5 (3.3–5.2) min  Mean procedure time: 104.231±13.468 (90–130) min  Prolonged ablation: 14 (54)  Antenna repositioned: 1 (4) | Session per patient NR  Mean treatment time: 2.5 (2-5) min | NR | Session per patient NR  MWA was conducted at 40W for 5–10 min |
| **Length of follow-up** | NR (reported outcomes immediately post ablation) | Median: 36 (13–47) months | 7-10 days | 3 months |
| **Loss to follow-up, n (%)** | NA | 0 | 0 | 0 |
| **Outcomes** | | | |  |
| **Efficacy** | | | |  |
| **Mortality** | 0 | 0 | 0 | 0 |
| **Complete ablation, n (%)** | Directly after procedure**:** 26 (100) | 1 week:  **Total:** 32 (91.4)  **MWA:** 15/15 (100)  **MWA+surgery:** 17/20 (85) | 7-10 days:  **Total:** 34/37* (91.9) (95%CI: 78.1-98.3)  **MWA:** 18/19 (94.7)  **MWA+cam**: 16/18 (88.8)  *reasons for not being able to assess whether complete ablation had been achieved included preoperative treatment (n = 1) and absence of intraoperative specimen analysis (n = 2) | 3 months:  13/18(72) |
| **Determination of complete ablation** | Histologic | **MWA:** imaging-based  **MWA+surgery:** histologic | Histologic | Histopathologic examination 3 months after ablation (tumour assessed with cytokeratin 8 and 18 and hematoxylineosin staining) |
| **Residual tumour, n (%)** | 0 (0) | **Total:** 3 (9)  **MWA:** 0 (0)  **MWA+surgery**: 3 (15) | **Total**: 3/37* (8.1)  **MWA:** 1/19 (5.2)  **MWA+cam:** 2/18 (1.1)  *reasons for not being able to assess whether complete ablation had been achieved included preoperative treatment (n = 1) and absence of intraoperative specimen analysis (n = 2) | 5 (28) |
| **Recurrence, n (%)** | NR | Local recurrence: median FU: 13-47m  **MWA:** 0 (0)  **MWA+surgery**: NR | NR | NR |
| **Cosmetic results, n/N (%)** | **Cometic satisfaction**:  patients satisfied: 26 (100) | NR | NR | Overall cosmetic outcomes were rated as good after thermal ablation (not reported per study arm) and intermediate after surgery (BCTOS median 1.6 vs 1.8). BCCT.core rated 94% of cases as good/excellent after thermal ablation versus 80% after surgery. |
| **Quality of life** | NR | NR | NR | BREAST-Q psychosocial, sexual well-being and satisfaction domains were generally higher after thermal ablation (not reported per study arm) than after surgery. |
| **Safety** | | | |  |
| **Adverse events (overall), n (%)** | **During procedure:**   - Slight pain requiring administration of lidocaine mixed with saline: 3 (12) - Subtle heat/swelling: 11 (42)   **Post procedure:**   - Moderate pain: 1 (4) - Postoperative oozing/other complication: 0 - Induration due to swelling and fibrosis around ablation area: 16 (62) | **During procedure:**   - 35 (100) showed local swelling at the treatment site about 2–3 days after ablation which then disappeared in 1 week - 2 (5.7) suffered moderate pain in the procedure of ablation, and the prescheduled ablation was completed after additional local anaesthesia. | MWA-related AEs  *Total* (n=40):   - pain during MWA: 7 (17.5) - local skin burn: 4 (10) - local skin necrosis: 1 (2.5) - poor incision healing after surgery: 2 (5)   *Grade 1***:**   - pain during MWA: 1 (2.5) - Local skin burn: 4 (10)   *Grade 2***:**   - pain during MWA: 6 (15) - local skin necrosis: 1 (2.5) - poor incision healing after surgery: 2 (5) | MWA-related AEs  Skin burn: 6 (33)  Oncoplastic surgery: 2 (11) |
| **Serious adverse events** | 0 | 0 | 0 | 0 |

*Abbreviations: AE – adverse event, BC – breast cancer, BCCT.core - Breast Cancer Conservative Treatment. Cosmetic results, BCS – breast-conserving surgery, BCTOS – Breast Breast Cancer Treatment Outcome Scale, CAM – complementary and alternative medicine, CI – confidence interval, DCIS – ductal carcinoma in situ, ER – oestrogen receptor, FU – follow-up, HER2 – human epidermal growth factor receptor 2, HR – hazard ratio / hormone receptor, Hx – history, IDC – invasive ductal carcinoma, ILC – invasive lobular carcinoma, MWA – microwave ablation, MST – mastectomy, MRI – magnetic resonance imaging, NA – not applicable, NR – not reported, PR – progesterone receptor, RCT – randomized controlled trial, SD – standard deviation, TNM – tumour-node-metastasis staging, Tx – treatment, US – ultrasound*

Table A - 8: Radiofrequency ablation: Results from randomised controlled trials

| **Author, year** | **Garcia-Tejedor, 2018 [51]** |
| --- | --- |
| **Country** | Spain |
| **Sponsor** | NR |
| **Intervention, Product** | RFA, Covidien (Tyco Healthcare Group, Bolder, USA) |
| **Comparator** | Surgery, lumpectomy |
| **Study design** | Phase II RCT, prospective, single centre, open label |
| **Primary study endpoints** | Intraoperative free margins (distance between the tumour and the margin in order to indicate if extensions are mandatory) |
| **Guidance, n (%)** | US |
| **Resection, n (%)** | **Yes**, immediately after ablation,   - lumpectomy: 20 (100) vs 20 (100) |
| **(Neo)Adjuvant Therapy, n (%)** | Neoadjuvant**:** 0 (0) vs 0 (0) (exclusion criteria)  Adjuvant**:**   - Endocrine: 19 (95) vs 18 (90), p=0.99 - Chemo: 8 (40) vs 5 (25), p=0.31 - Partial breast irradiation: 3 (15) vs 9 (45), p=0.038 - Lymph node irradiation: 3 (15) vs 1 (5), p=0.61 |
| **Anaesthesia during ablation, n/N (%)** | General |
| **Inclusion criteria** | - Female, >40 years - IDC ≤2.0 cm - ≤20% intraductal component - Tumour to skin and chest wall surface ≥1.0 cm. |
| **Exclusion criteria** | - Male gender - <40 years - Multifocal tumours - Extensive intraductal component neoadjuvant Tx - Previous surgery or radiation of the ipsilateral breast - Pregnancy or breast feeding. |
| **Recruitment period** | 09.2013 - 02.2017 |
| **Number of patients** | 40 (20 vs 20) |
| **Number of tumours** | 40 (20 vs 20) |
| **Age of patients, mean/median ± SD (range)** | - overall: 64 (46-86) - mean: 64 vs 64 - median: 64 vs 64 |
| **Sex, menopausal status, n (%)** | Female: 20 vs 20   - Postmenopausal: 18/20 (90) vs 18/20 (90) - Premenopausal: 2/20 (10) vs 2/20 (10) |
| **BC type, n (%)** | **Tumour grade**   - G1: 10 (50) vs 11 (55) - G2: 7 (35) vs 5 (25) - G3: 3 (15) vs 4 (20)   **Receptor status**   - ER+/PR+: 11 (55) vs 11 (55) - HER2-: 7 (35) vs 6 (30) - HER2+: 1 (5) vs 1 (5) - HER2 enriched: 1 (5) vs 1 (5) - Triple negative: 0 (0) vs 1 (5) |
| **Size of tumour (mm),**  **mean/median ± SD (range) / n (%)** | Median:   - radio: 13 vs 10.5, p=0.33 - pathology: 11.5 (5-20) vs 10.5 (6-16), p=0.07 |
| **Number of sessions in number of patients** | 1 session per patient, 8-10 min |
| **Length of follow-up** | - Immediately after surgery, 15 days, 6, 12, 18 months, 2 years - Median: 26.3 vs 23.7 months, p=0.58 |
| **Loss to follow-up, n (%)** | 0 (0) vs 0 (0) |
| **Outcomes** | |
| **Efficacy** | |
| **Mortality** | Median FU of 25 months (range 1-83): 0/20 (0) vs 0/20 (0) |
| **Complete ablation, n (%)** | RFA: 20 (100) |
| **Determination of complete ablation** | Histologic |
| **Residual tumour, n (%)** | NR |
| **Recurrence, n (%)** | After median FU of 25 months (range 1-83):   - local recurrence: 0 vs 0 - distant metastasis: 0 vs 0 |
| **Cosmetic results, n/N (%)** | Cosmetic satisfaction:  Good or very good: 17 (85) vs 17 (85), p=0.56 |
| **Quality of life** | NR |
| **Safety** | |
| **Adverse events (overall), n (%)** | Local adverse effects **after surgery**: 8 (40) vs 1 (5), p<0.01   - Breast inflammation: 5 (25) vs 1 (5), p=0.18 - Breast infection**:** 3 (15) vs 0 (0), p=0.23   * Recruitment was stopped with 20 participants in each group after the pre-planned interim analysis because of the higher amount of local adverse effects observed in the RFA arm. |
| **Serious adverse events** | NR |

*Abbreviations: BC – breast cancer, ER – oestrogen receptor, FU – follow-up, G1, G2, G3 – tumour grades 1, 2, 3, HER2 – human epidermal growth factor receptor 2, IDC – invasive ductal carcinoma, NR – not reported, PR – progesterone receptor, RCT – randomized controlled trial, RFA – radiofrequency ablation, Tx – treatment, US – ultrasound*

Table A - 9: Radiofrequency ablation: Results from single-arm trials

| **Author, year** | **Schassburger, 2014 [52]** | **Kinoshita, 2026, RAFAELO study (NCCH1409) [53]** |
| --- | --- | --- |
| **Country** | Sweden | Japan |
| **Sponsor** | Swedish BC Association, Capio Research Foundation, AFA Insurance, NeoDynamics AB, Bracco Diagnostics | Grant from the Japan Agency for Medical Research and Development (AMED): JP16lk0201029, JP19ck0106318, and JP23ck0106590 |
| **Intervention, Product** | RFA, Neodynamics, AB, Sweden | Cool-tip RF System or Cool-tip RF ablation System E Series (Medtronic plc, MN, USA) |
| **Comparator** | None | None |
| **Study design** | Single-arm | Multicentre, phase 3, single-arm study |
| **Primary study endpoints** | Treatment efficacy and safety | 5-year ipsilateral breast tumour recurrence-free survival (IBTRFS) rate in the full analysis set |
| **Guidance, n (%)** | US | US |
| **Resection, n (%)** | **Yes**, median days after ablation: 14.5 (6-22),  BCS: 17 (94)  MST: 1 (6) | **No** **routine** resection, **only if suspected residual tumour** from biopsy or imaging.   - partial MST: 13 patients |
| **(Neo)Adjuvant Therapy, n (%)** | Neoadjuvant: NR  Adjuvant: 18 (100)   - Radiation: 17 (94) - Endocrine: 18 (100) | - Radiation: 353 (100% of followed-up patients) - After radiation, hormone receptor-positive patients received adjuvant pharmacotherapy, including - endocrine therapy: 328 (93), - chemotherapy: 38 (11), - molecular-targeted therapy: 13 (4) |
| **Anaesthesia during ablation, n/N (%)** | - Local: 18/18 (100) - Additional light sedation: 2/18 (11) | - General |
| **Inclusion criteria** | - Unifocal BC ≤2.0 cm - ≤25% intraductal component - ER+, PR+, HER2+, tumour grade 3 | - Female patients aged 20–79 years with a single breast   cancer lesion classified as Tis–T1 (tumour size ≤ 1.5 cm),   - N0M0 stage 0–I, - Able to tolerate postoperative radiotherapy, chemotherapy, and endocrine therapy |
| **Exclusion criteria** | - Multifocal tumours - Diffuse growth pattern - DCIS - Lobular cancer | - Extensive intraductal breast lesions or - Suspected multiple lesions on imaging |
| **Recruitment period** | NR | 08.2013 – 11.2017 |
| **Number of patients** | 18 | 370 |
| **Number of tumours** | 18 | 370 |
| **Age of patients, mean/median ± SD (range)** | Median: 67 (46-84) | Median 55 (47–65) |
| **Sex, menopausal status, n (%)** | Female,   - Postmenopausal: 17/18 (94) - Premenopausal: 1/18 (6) | Female, NR |
| **BC type, n (%)** | **Histology**   - Ductal: 15 (83) - Ductal/Tubular: 1 (6) - Tubular: 2 (11)   **Tumour grade**   - G1: 5 (28) - G2: 13 (72) - G3: 0 (0)   **Receptor status**   - ER+: 18 (100) - PR+: 16 (89), PR- 2 (11) - HER2-: 15 (83) - HER2+: 3 (17) | **Histology on biopsy**   - IDC: 307 (87) - DCIS: 40 (11) - DC: 6 (2)   **Histological grade**   - 1: 163 (46) - 2: 102 (29) - 3: 17 (5) - Unknown: 71 (20)   **Receptor status**   - ER+: 338 (96) - PR+: 314 (89) - HER2−: 319 (90) |
| **Size of tumour (mm),**  **mean/median ± SD (range) / n (%)** | Median:   - MRI: 11 (5-20) - US: 10 (6-15) - Mammography: 10 (6-15) - Pathology: 10 (5-16) | Median:   - US: 10 (8–12) - MRI: 11 (9–13)   Mean:   - US: 9.8 (2.8) - MRI: 10.7 (2.8) |
| **Number of sessions in number of patients** | 1 session per patient, median time: 10 (8-14) min | 1 session per patient, mediant treatment time 7.5 min (interquartile range, 6–10), mean treatment time 8.4 min (3.6) |
| **Length of follow-up** | Median: 14.5 (6-22) days | Median: 5.0 (IQR 5.0–5.1) years |
| **Loss to follow-up, n (%)** | 0 | 17 |
| **Outcomes** | | |
| **Efficacy** | | |
| **Mortality** | 0 | All-cause: 4 (1)   - Breast cancer-related: 2 - Due to other diseases: 2   5-year OS: 99.2% (95% CI 97.4–99.7)  5-year metastasis-free survival: 99.2% (97.4–99.7)  5-year IBTRFS rate: 98.6% (95% CI 96.6–99.4) |
| **Complete ablation, n (%)** | MRI: median FU of 13 days: 18 (100)  Resection: median: 14.5 (6-22) days: 15/18 (83); 16/18 (85); 18 (100) with at least one histological staining method | **3 months**: 342 (97) |
| **Determination of complete ablation** | Imaging and histologic | Pathological examination of samples obtained by vacuum-assisted breast biopsy (VAB) was conducted 3 months after radiation therapy for 352/353 patients. This analysis involved hematoxylin and eosin staining as well as nicotinamide adenine dinucleotide staining or single-strand DNA staining. |
| **Residual tumour, n (%)** | Median FU of 13 days: 0 (0) | **5 years**: 10 (3)*  *was considered to represent incomplete ablation and not classified as recurrence. |
| **Recurrence, n (%)** | NR | 2 (1) in the ipsilateral breast  5-year cumulative ipsilateral breast tumour recurrence rate: 0.6% (95% CI 0.1–1.9) |
| **Cosmetic results, n/N (%)** | NR | NR |
| **Quality of life** | NR | NR |
| **Safety** | | |
| **Adverse events (overall), n (%)** | Own pain assessment/management protocol using VAS (0=no pain, 10= unbearable pain):   - Pain during administration of anaesthetics: 2 VAS - Pain during procedure: 2.5 VAS, p=0.512 - Median pain before procedure: 0 - Median pain after procedure: 0.5, p=0.042 | AEs observed **during RFA**:  Thermal burn *Grade 1 + Grade 2*: 7  AEs observed from **after RFA to the start of radiation**:  *Grade 1*: 17  *Grade 2*: 1  *≥ Grade 3*: 0 (0) |
| **Serious adverse events** | NR | AEs observed from **after RFA to the start of radiation**:  Skin ulcer Grade 3: 1 (0.3)  Grade 4: 0 (0) |

*Abbreviations: BC – breast cancer, BCS – breast-conserving surgery, DCIS – ductal carcinoma in situ, ER – oestrogen receptor, FU – follow-up, G1, G2, G3 – tumour grades 1, 2, 3, HER2 – human epidermal growth factor receptor 2, IDC – invasive ductal carcinoma, IBTRFS - ipsilateral breast tumour recurrence-free survival*, *IQR – interquartile range, MST – mastectomy, MRI – magnetic resonance imaging, NR – not reported, PR – progesterone receptor, RCT – randomised controlled trial, RFA – radiofrequency ablation, Tx – treatment, US – ultrasound, VAB – vacuum-assisted breast biopsy, VAS – visual analogue scale*

Table A - 10: High-intensity focused ultrasound ablation: Results from single-arm trials

| **Author, year** | **Guan, 2016 [54]** | **Merckel, 2016 [55]** |
| --- | --- | --- |
| **Country** | China | Netherlands |
| **Sponsor** | None | Center for Translational Molecular Medicine |
| **Intervention - Product** | HIFU, JC tumour treatment system (Haifu Medical Technology co., Ltd, Chongqing, China); PZT-4 piezo-ceramic ultrasound transducer (Beijing Cheng-Cheng Weiye Science and Technology Co., Ltd, Beijing, China); AU3 ultrasound imaging device (Esaote, Genoa, Italy) | HIFU, Sonalleve-based prototype (Philips Healthcare, Vantaa, Finland) |
| **Comparator** | Surgery, modified radical mastectomy without any other treatment before surgery | None |
| **Study design** | Prospective, single-arm*  *Originally an RCT | Prospective, single-arm |
| **Primary study endpoint** | Damage effect of HIFU on BC tissues and their vascularities | Treatment feasibility and safety |
| **Guidance** | US | MRI |
| **Resection, n (%)** | **Yes,** 1-2 weeks after ablation, modified radical mastectomy: 25 (100) | **Yes**, 48h-10 days after ablation,   - lumpectomy: 8 (80) - MST: 1 (10) - No surgery: 1 (10) |
| **(Neo)Adjuvant Therapy, n (%)** | - Neoadjuvant**:** NR - Adjuvant**:** 25 (100) vs 25 (100) | - Neoadjuvant**:** 0 (0) (exclusion criteria) - Adjuvant**:** NR |
| **Anesthesia during ablation, n/N (%)** | General | Procedural sedation |
| **Inclusion criteria** | - Solitary invasive BC (T1-2, N0-2, M0) ≤5.0 cm - Tumour to skin surface/ribcage ≥1.0 cm - Tumour to nipple ≥2.0 cm | - Female, ≥18 years - Invasive BC (T1-2) ≤1.0 cm - Tumour to skin surface and pectoralis ≥1.0 cm and within reach of the - HIFU transducers with the patient in prone position - WHO performance status ≤2, weight ≤80 kg |
| **Exclusion criteria** | - Multifocal tumours or tumours with undefined margins - Coagulation disorders, myocardial disease or diabetes | - Neoadjuvant Tx - C/I for MRI - Macro-calcifications scar tissue or surgical clips in path of the ultrasound beams |
| **Recruitment period** | 02.2014 - 08.2014 | 09.2012 - 06.2014 |
| **Number of patients** | 50 (25 vs 25) | 10 |
| **Number of tumours** | 50 (25 vs 25) | 10 |
| **Age of patients, mean/median±SD (range)** | Mean: 48 (22-63) vs 45 (25-65) | Mean: 54.8±12.5 |
| **Sex, menopausal status** | Female,   - Premenopausal: 5 (20) vs 4 (16) - Peri-menopausal: 5 (20) vs 4 (16) - Postmenopausal: 16 (64) vs 16 (64) | Female, NR |
| **BC type, n (%)** | **Histology,** p=NS   - IDC: 13 (52) vs 14 (56) - ILC: 7 (28) vs 5 (20) - other: 5 (20) vs 6 (24)   **TNM Staging,** p=NS   - Stage 1: 6 (24) vs 7 (28) - Stage 2A: 4 (16) vs 5 (20) - Stage 2B: 15 (60) vs 13 (52)   **Tumour grade**   - G1: 10 (40) vs 12 (48) (p=NS) - G2: 12 (48) vs 9 (36) - G3: 3 (12) vs 4 (16)   **Receptor status,** p=NS   - ER+, PR+: 13 (52) vs 14 (56) - ER+, PR−: 4 (16) vs 2 (8) - ER-, PR−: 7 (28) vs 8 (32) | **Histology**   - IDC: 8 (80) - ILC: 2 (20) |
| **Size of tumour (mm),**  **mean/median±SD (range) / n (%)** | (21-48) vs (23-45) | Mean: Pathology: 20.0±5.6 |
| **Number of sessions in number of patients** | NR | Mean treatment time: 145 min  Actual sonification time: 1.7 min |
| **Length of follow-up** | 2 weeks, 12 months   - median: 368 days - mean: 12 months | 48h-10 days |
| **Loss to follow-up, n (%)** | 2 weeks: 0 (0) vs 0 (0)  12 months: 0 (0) vs 0 (0) | 0 |
| **Outcomes** | | |
| **Efficacy** | | |
| **Mortality** | 0 vs 0 | 0 |
| **Complete ablation, n (%)** | 1-2 weeks: 25 (100) | NA  *only reported on the extent of tumour necrosis, not evident whether the extend describes full ablation |
| **Determination of complete ablation** | Histologic | Histologic |
| **Residual tumour, n (%)** | 0 (0) | NA |
| **Recurrence, n (%)** | 12 months: 0 vs 0 | NR |
| **Cosmetic results, n/N (%)** | NR | NR |
| **Quality of life** | NR | NR |
| **Safety** | | |
| **Adverse events (overall), n (%)** | **Post procedure**:   - oedema in the mammary tissue circumjacent the ablated tumour: 25 (100) - pain, tenderness, discomfort: 11 (44) - mild fever: 3 (12) | **48 hours - 10 days:**  Minor AEs: 5   - nausea and vomiting: 2 (20) - pain: 2 (20)* - skin changes: 1 (10)   *Scores 4 and 5 out of 10 |
| **Serious adverse events** | NR | 0 |

*Abbreviations: AE – adverse event, BC – breast cancer, C/I – contraindication, DCIS – ductal carcinoma in situ, ER – oestrogen receptor, FU – follow-up, G1, G2, G3 – tumour grades 1, 2, 3, HIFU – high-intensity focused ultrasound, IDC – invasive ductal carcinoma, ILC – invasive lobular carcinoma, MST – mastectomy, MRI – magnetic resonance imaging, NA – not applicable, NR – not reported, NS – not significant, PR – progesterone receptor, RCT – randomised controlled trial, RFA – radiofrequency ablation, SD – standard deviation, TNM – tumour, node, metastasis (staging system), Tx – treatment, US – ultrasound, WHO – World Health Organization*

Table A - 11: Laser ablation: Results from single-arm trials

| **Author, year** | **Schwartzberg, 2018 [56]** |
| --- | --- |
| **Country** | USA, UK |
| **Sponsor** | Novian Health |
| **Intervention, Product** | LA, Novilase Laser Therapy (Novian Health, Chicago, IL, USA) |
| **Comparator** | None |
| **Study design** | Single-arm - prospective, multicenter, open label |
| **Primary study endpoint** | Rate of complete tumour ablation |
| **Guidance, n (%)** | - Ultrasound: 60 (98) - Stereotaxis: 1 (2) |
| **Resection, n (%)** | **Yes**, within 28 days after ablation:   - Lumpectomy: 55 (90) - MST: 6 (10) |
| **(Neo)Adjuvant Therapy, n (%)** | Neoadjuvant: 0 (0) (exclusion criteria)  Adjuvant: 1 (2)   - Chemo + radiation: 1 (2) |
| **Anaesthesia during ablation, n/N (%)** | Local |
| **Inclusion criteria** | - Female, 18-80 years - Unifocal IDC ≤2.0 cm - ≤25% intraductal components - Tumour to skin and chest wall surface ≥0.5 cm. |
| **Exclusion criteria** | - Benign tumours and DCIS - BRCA + - Neoadjuvant treatment - Hx of PLA BC Tx - Recurrent BC - Morbid obesity, renal insufficiency and other comorbidities affecting life expectancy - Pacemakers/metallic implants - Pregnancy or breast feeding. |
| **Recruitment period** | 06.2012 - 05.2015 |
| **Number of patients** | 61 |
| **Number of tumours** | 61 |
| **Age of patients, mean/median ± SD (range)** | Mean: 64 (42-77) |
| **Sex, menopausal status** | Female, NR |
| **BC type, n (%)** | **Histology**   - IDC: 47 (77) - Infiltrating ductal /ductal in situ: 9 (14.7) - DCIS: 1 (1.6) - other: 3 (4.9)   **Tumour grade**   - G1: 24 (39.3) - G2: 31 (50.8) - G3: 6 (9.8)   **Tumour subtype**   - HER2-, ER+: 50 (81.9) - HER2+, ER+: 4 (6.5) - HER2+, ER-: 2 (3.3) - HER2 equivocal: 2 (3.3) - HER2-, ER-: 1 (1.6) - Unknown: 2 (3.3) |
| **Size of tumour (mm),**  **mean/median ± SD (range) / n (%)** | Mean:  MRI: 11.3 (4.0-19.0) |
| **Number of sessions in number of patients** | 1 session per patient;  Mean laser time: 15.8 (14.5-36.5) min;  Average time of total procedure <1 hour |
| **Length of follow-up** | Surgical excision within 28 days,  FU up to 5 years  Mean: 43 (34-65) months |
| **Loss to follow-up, n (%)** | NR |
| **Outcomes** | |
| **Efficacy** | |
| **Mortality** | NR |
| **Complete ablation, n (%)** | 28 days: 51 (84) |
| **Determination of complete ablation** | Histology |
| **Residual tumour, n (%)** | 28 days: 10 (16) |
| **Recurrence, n (%)** | 4 years: 2 (3) |
| **Cosmetic results, n/N (%)** | 28 days: Cosmetic satisfaction (measured by questionnaire):   - Excellent: 27/58 (64) - Good: 19/58 (33) - <Good: 12/58 (21) - Missing: 3/61 (5) - Significant scar: 1 (2) |
| **Quality of life** | Health-related quality-of-life (EORTC QLQ-BR23 and QLQ-C30 surveys):  Change of ≥5 points compared with reference mean for early-stage BC: 100% of patients |
| **Safety** | |
| **Adverse events (overall), n (%)** | Mean **FU: 43 (34-65) months**:  *Mild AEs*: 8   - Lump: 1 (2) - Blister: 2 (3) - Hematoma: 1 (2) - Erythema: 1 (2) - Fat necrosis: 3 (5)   *Moderate AEs*: 6   - Pain: 4 (7) - Lump: 1 (2) - Seroma: 1 (2)   Average maximum pain during treatment: 4.2±2.9 (0-10) |
| **Serious adverse events** | 0 |

*Abbreviations: AE – adverse event, BC – breast cancer, BRCA+ – mutation in BRCA gene (breast cancer susceptibility gene), DCIS – ductal carcinoma in situ, ER – oestrogen receptor, EORTC QLQ-BR23 – European Organisation for Research and Treatment of Cancer Quality of Life Questionnaire - Breast Cancer Module, EORTC QLQ-C30 – European Organisation for Research and Treatment of Cancer Quality of Life Questionnaire - Core 30, FU – follow-up, G1, G2, G3 – tumour grades 1, 2, 3, HER2 – human epidermal growth factor receptor 2, Hx – history, IDC – invasive ductal carcinoma, ILC – invasive lobular carcinoma, LA – laser ablation, MST – mastectomy, MRI – magnetic resonance imaging, NR – not reported, NS – not significant, PLA – percutaneous laser ablation, PR – progesterone receptor, QLQ – quality of life questionnaire, Tx – treatment, UK – United Kingdom, USA – United States of America*

Risk of Bias Tables

Table A - 12: ROB2 of RCTs comparing thermal ablation techniques with surgical resection

| **Trial** | **Endpoints** | **Bias arising from the randomization process** | **Bias due to deviations from intended interventions** | **Bias due to missing outcome data** | **Bias in measurement of the outcome** | **Bias in selection of the reported result** | **Overall risk of bias** |
| --- | --- | --- | --- | --- | --- | --- | --- |
| **CYA** | | | | | | | |
| No RCTs identified | | | | | | | |
| **MWA** | | | | | | | |
| No RCTs identified | | | | | | | |
| **RFA** | | | | | | | |
| Garcia-Tejedor, 2018 [51] | Mortality | Low | Low | Low | Low | Low | Low |
|  | Complete tumour ablation | Low | Low | Low | Low | Low | Low |
|  | Recurrence | Low | Low | Low | Low | Low | Low |
|  | Safety | Low | Low | Low | Low | Low | Low |
| **HIFU** | | | | | | | |
| No RCTs identified | | | | | | | |
| **LA** | | | | | | | |
| No RCTs identified | | | | | | | |

*Abbreviations:* *CYA – cryoablation, HIFU – high-intensity focused ultrasound ablation, LA – laser ablation, MWA – microwave ablation, RCT– randomized controlled trial, RFA* *– radiofrequency ablation, RoB – risk of bias*

Table A - 13: Outcome-specific ROBINS-I of NRSI comparing thermal ablation techniques with surgical resection

| **Study  reference/ID** | **Outcome** | **Bias due to confounding** | **Bias selection of participants into the study** | **Bias in measurement of intervention** | **Bias due to departures from intended interventions** | **Bias due to missing data** | **Bias in measurement of outcomes** | **Bias in selection of the reported results** | **Overall bias** | **Comments** |
| --- | --- | --- | --- | --- | --- | --- | --- | --- | --- | --- |
| **CYA** | | | | | | | | | | |
| Galati, 2024 [33] | Adverse events | Critical^a^ | - | - | - | - | - | - | Critical | Only adverse events were described for both groups. |
| **MWA** | | | | | | | | | | |
| Zhong, 2023 [47] | Mortality | Serious^b^ | Low | Low | Low | Serious^c^ | Low | Low | Serious | Only OS, DFS and recurrence were described for both groups. |
|  | Disease free survival | Serious^b^ | Low | Low | Low | Serious^c^ | Low | Low | Serious |  |
|  | Recurrence | Serious^b^ | Low | Low | Low | Serious^c^ | Low | Low | Serious |  |
| **RFA** | | | | | | | | | | |
| No NRSI identified | | | | | | | | | | |
| **HIFU** | | | | | | | | | | |
| No NRSI identified | | | | | | | | | | |
| **LA** | | | | | | | | | | |
| No NRSI identified | | | | | | | | | | |

*Abbreviations: CYA – cryoablation, DFS – disease free survival, HIFU – high-intensity focused ultrasound ablation, LA – laser ablation, MWA – microwave ablation, NRSI –non-randomised study of intervention, OS – overall survival, RFA – radiofrequency ablation, RoB – Risk of Bias*

*Comments:*

*^a^ According to the guideline of the newest ROBINS-I tool, no further assessment was conducted after finding the domain “bias due to confounding” critical.*

*^b^ Only patients in the surgery group underwent axillary management (p=0.001). This could have influenced tumour recurrence rates and overall survival.*

*^c^ Concerns regarding missing patients at later follow-up dates without details on the management of missing data.*

GRADE Evidence Profiles

Table A - 14: Evidence profile: efficacy and safety of cryoablation in early-stage breast cancer

| **Quality assessment** | | | | | | | **Summary of findings** | | | | |
| --- | --- | --- | --- | --- | --- | --- | --- | --- | --- | --- | --- |
|  |  |  |  |  |  |  | **Number of patients** | | **Effect** | | **Quality** |
| **Number  of studies** | **Study design** | **Risk of bias** | **Inconsistency** | **Indirectness** | **Imprecision** | **Other  considerations** | **CYA** | **Surgery** | **Relative (95% CI)** | **Absolute (95% CI)** |  |
| **EFFICACY** | | | | | | | | | | | |
| **Overall survival (follow-up: 1 month to 73 months)** | | | | | | | | | | | |
| 10 [34-41, 43, 44, 46] | Single-arm | Serious^a^ | Not serious | Not serious | Serious^b^ | None | 503 | NA | FU 1 m - 73 m:  475/503 (94%; median: 100%; range: 89% to 100%)  FU 1 - 12 m:  192/194 (99%)  FU >12 - 73 m:  283/309 (92%) | | ⨁◯◯◯  Very low |
| **Disease-free survival – not reported** | | | | | | | | | | | |
| 0 | NA | NA | NA | NA | NA | NA | NA | NA | NA | NA | NA |
| **Complete tumour ablation** | | | | | | | | | | | |
| 1 [33] | NRSI | Very serious^c^ | Not serious | Not serious | Not serious | None | 10 | NA | 9/10 (90%) vs NA | | ⨁◯◯◯  Very low |
| 7 [37, 40-44, 46] | Single-arm | Serious^a^ | Not serious | Not serious | Not serious | None | 293 | NA | 270/293 (92%; median 92; range: 53% to 99%) | | ⨁⨁◯◯ Low |
| **Recurrence/local and distant (follow-up: 18 to 60 months)** | | | | | | | | | | | |
| 6 [34-39, 44] | Single-arm | Serious^a^ | Not serious^d^ | Not serious | Not serious | None | 364 | NA | FU 18 m – 60 m:  20/364 (5%; median: 3%; range: 0% to 22%) | | ⨁⨁◯◯ Low |
| **SAFETY** | | | | | | | | | | | |
| **Adverse events (Immediate/post procedure)** | | | | | | | | | | | |
| 1 [33] | NRSI | Very serious^c^ | Not serious | Not serious | Serious^e^ | None | 10 | 10 | Minor AE:  2/10* vs 0/10  *small post-ablative hematoma (about 4 cm)  Pain (assessed on a scale from 1-10):  Median score: 3 vs 5, p=NR | | ⨁◯◯◯  Very low |
| 5 [34, 37, 40-42] | Single-arm | Serious^a^ | Serious^f^ | Not serious | Serious^e^ | None | 129 | NA | 39/129 (30%; median 22%; range: 0 to 100)  Most frequent AEs: mild to moderate pain (16/39 AEs, 41%) and bruising: (29/39 AEs, 74%) | | ⨁◯◯◯  Very low |
| **Adverse events (follow-up: 1 week to 5 years)** | | | | | | | | | | | |
| 1 [33] | NRSI | Very serious^c^ | Not serious | Not serious | Serious^e^ | None | 10 | 10 | FU 1 w: 0 vs 0 | | ⨁◯◯◯  Very low |
| 8 [34-38, 40, 41, 44]* | Single-arm | Serious^a^ | Serious^f^ | Not serious | Not serious | None | 385 | NA | FU up to 2 w: 0% to 6% patients [37, 38],  FU 2 w: 79 AEs in 105 patients [41, 44]  FU 3 m: 4% patients [34]  FU 6 m: 49 AEs in 85 patients [44]  During FU 3 yrs: 43 AEs in 6% patients, during 5 yrs 187 events in 50% patients [35, 36] | | ⨁◯◯◯  Very low |
| **Serious adverse events (follow-up: 1 week to 5 years)** | | | | | | | | | | | |
| 1 [33] | NRSI | Very serious^c^ | Not serious | Not serious | Serious^e^ | None | 10 | 10 | FU 1 w: 0 vs 0 | | ⨁◯◯◯  Very low |
| 5 [34-38, 46] | Single-arm | Serious^a^ | Not serious | Not serious | Not serious | None | 265 | NA | FU 2 m – 60 m: 0 (0) | | ⨁◯◯◯  Very low |

*Abbreviations: AE – adverse event, CI – confidence interval, CYA – cryoablation, FU– follow-up, m – months, NA – not applicable, NRSI –- non-randomized study of intervention, RoB – Risk of Bias, vs – versus*

*Comments: ** 2 studies [41, 44] reported AEs as event categories rather than unique affected patients and could not be pooled quantitatively.

*^a^ According to the HTA-Guidelines and LATITUDES recommended risk of bias tools, single-arm trials were considered to have a high risk of bias.*

*^b^ Most deaths (n=21) reported in one study with the longest FU [Fine, 2024], of which 16 were unrelated to BC and 3 for unknown reasons.*

*^c^ Assessed as having a critical risk of bias, confounding factors were not adjusted for.*

*^d^ High recurrence rate in study with 23 patients, tumour sizes bigger than in other studies and included patients, who were “unsuitable for surgery” [34].*

*^e^ Very low sample size, OIS not reached.*

*^f^ High variation in what and how adverse events were reported.*

Table A - 15: Evidence profile: efficacy and safety of microwave ablation in early-stage breast cancer

| **Quality assessment** | | | | | | | **Summary of findings** | | | |
| --- | --- | --- | --- | --- | --- | --- | --- | --- | --- | --- |
|  |  |  |  |  |  |  | **Number of patients** | | **Effect (I vs C)** | **Quality** |
| **Number  of studies** | **Study design** | **Risk of bias** | **Inconsistency** | **Indirectness** | **Imprecision** | **Other  considerations** | **MWA** | **Surgical Resection** |  |  |
| **EFFICACY** | | | | | | | | | | |
| **Overall survival/Mortality (follow-up: immediately post ablation to 36 months)** | | | | | | | | | | |
| 1 [47] | Propensity score matched NRSI | Serious^a^ | Not serious | Serious^b^ | Serious^c^ | None | 33 | 99 | OS mean FU 31 m (2-74):  33 (100) vs 98 (99)  HR: 0.537  95%CI: 0.089-3.325, p=0.49  OS rate 1 y:  97% vs 100%  OS rate 3 y:  93% vs 96% | ⨁◯◯◯  Very low |
| 4 [46, 48-50] | Single-arm | Serious^d^ | Not serious | Not serious | Serious^e^ | None | 119 | NA | FU immediately post ablation to 36 m:  119 (100) | ⨁◯◯◯ Very low |
| **Disease-free survival** | | | | | | | | | | |
| 1 [47] | Propensity score matched NRSI | Serious^a^ | Not serious | Serious^b^ | Serious^c^ | None | 33 | 99 | Median FU  31 m (2-74):  HR: 0.536  95% CI: 0.128-2.249, p=0.38 | ⨁◯◯◯ Very low |
| 0 | Single-arm | NA | NA | NA | NA | NA | NA | NA | NA | NA |
| **Complete tumour ablation** | | | | | | | | | | |
| 1 [47]^f^ | Propensity score matched NRSI | Serious^d^ | Not serious | Serious^b^ | Serious^e^ | None | 33 | NA | FU 1 w: 32 (97)  FU 1 m: 33 (100),  95% CI: 89.4-100% | ⨁◯◯◯ Very low |
| 4 [46, 48-50] | Single-arm | Serious^d^ | Not serious | Not serious | Serious^e^ | None | 119 | NA | FU immediately post ablation to 10 days: 92/98 (94)  FU 3 m: 13/18 (72) | ⨁◯◯◯ Very low |
| **Recurrence/local and distant** | | | | | | | | | | |
| 1 [47] | Propensity score matched NRSI | Serious^g^ | Not serious | Serious^b^ | Serious^c^ | None | 33 | 99 | median FU: 31 m (2-74)  Local recurrence:  1 (3) vs 1 (1)  Distant metastasis:  0 (0) vs 2 (2) | ⨁◯◯◯ Very low |
| 1 [50] | Single-arm | Serious^d^ | Not serious | Not serious | Very serious^e^ | None | 35 | NA | Median FU 13-47m  MWA: 0/15 (0)  MWA+surgery: NR | ⨁◯◯◯ Very low |
| **SAFETY** | | | | | | | | | | |
| **Adverse events** | | | | | | | | | | |
| 1 [47]^f^ | Propensity score matched NRSI | Serious^d^ | Not serious | Not serious | Serious^e^ | None | 33 | NA | Median FU 31 m (2-74):  0 vs NR | ⨁◯◯◯ Very low |
| 4 [46, 48-50] | Single-arm | Serious^d^ | Not serious | Not serious | Serious^e^ | None | 119 | NA | Pain during or after the procedure (4 studies): 6-18% of patients,  swelling during or after the procedure (2 studies): 42% and 100%,  skin burn (2 studies): 10% and 33%, skin necrosis (1 study): 3% of patients; need for oncoplastic surgery (1 study): 11% of patients. | ⨁◯◯◯  Very low |
| **Serious adverse events** | | | | | | | | | | |
| 1 [47]^f^ | Propensity score matched NRSI | Serious^d^ | Not serious | Not serious | Serious^e^ | None | 33 | NA | 0 vs NR | ⨁◯◯◯  Very low |
| 4 [46, 48-50] | Single-arm | Serious^d^ | Not serious | Not serious | Serious^e^ | None | 119 | NA | 0 | ⨁◯◯◯  Very low |

*Abbreviations: AE – adverse event, CI – confidence interval, DFS – disease free survival, FU– follow-up, m – months, HR – hazard ratio, HTA – health technology assessment, MRI- magnetic resonance imaging, MWA – microwave ablation, NA – not applicable, NR – not reported, NRSI – non-randomised study of intervention, OIS – optimal information size, OS – overall survival, RoB – Risk of Bias, vs – versus*

*Comments:*

*^a^ Concerns regarding missing data and confounding factors (no lymph node dissection/SLNB in intervention group).*

*^b^ This study included only participants >70 years.*

*^c^ Certainty in evidence lowered because of a small number of events leading to wide confidence intervals.*

*^d^ According to the HTA-Guidelines and LATITUDES recommended risk of bias tools, single-arm trials were considered to have a high risk of bias.*

*^e^ Small sample size, OIS not reached.*

*^f^ Zhong et al. compared only the outcomes of mortality, OS, DFS and recurrence between the groups, other outcomes are described only for the intervention group, therefore we utilize this data like findings from a single-arm trial.*

*^g^ Concerns regarding missing data and confounding factors (no lymph node dissection/SLNB in intervention group).*

Table A - 16: Evidence profile: efficacy and safety of radiofrequency ablation in early-stage breast cancer

| **Quality assessment** | | | | | | | **Summary of findings** | | | | |
| --- | --- | --- | --- | --- | --- | --- | --- | --- | --- | --- | --- |
|  |  |  |  |  |  |  | **Number of patients** | | **Effect** | | **Quality** |
| **Number  of studies** | **Study design** | **Risk of bias** | **Inconsistency** | **Indirectness** | **Imprecision** | **Other  considerations** | **intervention** | **comparison** | **Relative (95% CI)** | **Absolute (95% CI)** |  |
| **EFFICACY** | | | | | | | | | | | |
| **Overall survival (FU immediately post-surgery-5 years)** | | | | | | | | | | | |
| 1 [51] | RCT | Not serious | Not serious | Not serious | Very serious^a^ | None | 20 | 20 | Median FU of 25 m (range: 1 to 83):  20/20 (100) vs 20/20 (100) | | ⨁⨁◯◯  Low |
| 2 [52] | Single-arm | Serious^b^ | Not serious | Not serious | Serious^c^ | None | 388 | NA | Median FU 14.5 d  18/18 (100)  Median FU of 5 years: 366/370 (99) | | ⨁◯◯◯  Very low |
| **Disease free survival** | | | | | | | | | | | |
| 0 | RCT | NA | NA | NA | NA | NA | NA | NA | NA | | NA |
| 0 | Single-arm | NA | NA | NA | NA | NA | NA | NA | NA | | NA |
| **Complete tumour ablation (FU immediately post-surgery – 3 months)** | | | | | | | | | | | |
| 1 [51] | RCT | Not serious | Not serious | Not serious | Serious^c^ | None | 20 | NA | Immediately after ablation:  20 (100) | | ⨁⨁◯◯ Low |
| 2 [52] | Single-arm | Serious^b^ | Not serious | Not serious | Serious^c^ | None | 388 | NA | MRI:  Median FU of 13 d  18 (100)  Histology:  Median FU of 14.5 days (range: 6-22):  15/18 (83) – 16/18 (85)^d^  FU of 3 m: 342/388 (97) | | ⨁◯◯◯  Very low |
| **Recurrence/local and distant (FU 2-5 years)** | | | | | | | | | | | |
| 1 [51] | RCT | Not serious | Not serious | Not serious | Very serious^a^ | None | 20 | 20 | Median FU of 25 m (range: 1-83):  local: 0 vs 0  distant metastasis: 0 vs 0 | | ⨁⨁◯◯  Low |
| 1 [53] | Single-arm | Serious^b^ | Not serious | Not serious | Serious^c^ | None | 370 | NA | FU of 5 years:  2 (1) in the ipsilateral breast  5-year cumulative ipsilateral breast tumour recurrence rate: 0.6% (95% CI 0.1–1.9%) | | ⨁◯◯◯  Very low |
| **SAFETY** | | | | | | | | | | | |
| **Adverse events** | | | | | | | | | | | |
| 1 [51] | RCT | Not serious | Not serious | Not serious | Serious^a^ | None | 20 | 20 | Total AE after surgery^e^: 8 (40) vs 1 (5), p=0.1  Breast inflammation:  5 (25) vs 1 (5), p=0.18  Breast infection:  3 (15) vs 0 (0), p=0.23 | | ⨁⨁⨁◯  Moderate |
| 2 [52, 53] | Single-arm | Serious^b^ | Not serious | Not serious | Serious^c^ | None | 388 | NA | Pain during anaesthetic administration vs procedure: VAS 2.0 vs 2.5; p=0.512.  Pain before vs after procedure: VAS 0 vs 0.5; p=0.042.  Thermal burn (grade 1 + 2): 7  AEs from after RFA to start of adjuvant radiation:  Grade 1: 17  Grade 2: 1  Grade ≥ 3: 4 (1) | | ⨁◯◯◯  Very low |
| **Serious adverse events** | | | | | | | | | | | |
| 0 | RCT | NA | NA | NA | NA | NA | NA | NA | NA | | NA |
| 0 | Single-arm | NA | NA | NA | NA | NA | NA | NA | NA | | NA |

*Abbreviations: AE – adverse event, CI – confidence interval, FU– follow-up, m – months, MRI- magnetic resonance imaging, NA – not applicable, OIS – optimal information size, RCT – randomized controlled trial, RFA – radiofrequency ablation, RoB – Risk of Bias, HTA – health technology assessment, VAS – visual analogue scale, vs – versus*

*Comments:*

*^a^ This study was prematurely stopped due to higher number of local adverse events in the intervention group; hence OIS was not reached. Further, there is a low number of events.*

*^b^ According to the HTA-Guidelines and LATITUDES recommended risk of bias tools, single-arm trials are considered to have a high risk of bias.*

*^c^ Small sample size (n=18); IOS not met.*

*^d^ Complete ablation was assessed with MRI and histological staining. Complete tumour devitalization was indicated in 15/18 (83%) of patients as judged by H&E staining and in 16/18 (89%) of patients as judged by CK8 staining.*

*^e^ Tumour was immediately resected after ablation in the intervention group.*

Table A - 17: Evidence profile: Efficacy and safety of high-intensity focused ultrasound in early-stage breast cancer

| **Quality assessment** | | | | | | | **Summary of findings** | | | | |
| --- | --- | --- | --- | --- | --- | --- | --- | --- | --- | --- | --- |
|  |  |  |  |  |  |  | **Number of patients** | | **Effect** | | **Quality** |
| **Number  of studies** | **Study design** | **Risk of bias** | **Inconsistency** | **Indirectness** | **Imprecision** | **Other  considerations** | **intervention** | **comparison** | **Relative (95% CI)** | **Absolute (95% CI)** |  |
| **EFFICACY** | | | | | | | | | | | |
| **Overall survival (follow-up 10 days to 12 months)** | | | | | | | | | | | |
| 2 [54, 55] | Single-arm | Serious^a^ | Not serious | Not serious | Serious^b^ | None | 35 | NA | At 12 m [54]: 25/25 (100%)  At 10 d [55]: 10/10 (100%) | | ⨁◯◯◯  Very low |
| **Disease free survival** | | | | | | | | | | | |
| 0 | NA | NA | NA | NA | NA | NA | NA | NA | NA | | NA |
| **Complete tumour ablation/Necrosis/Residual tumour** | | | | | | | | | | | |
| 1 [54] | Single-arm | Serious^a^ | Not serious | Not serious | Serious^b^ | None | 25 | NA | 25/25 (100%) | | ⨁◯◯◯  Very low |
| **Recurrence/local and distant (follow-up 12 months)** | | | | | | | | | | | |
| 1 [54] | Single-arm | Serious^a^ | Not serious | Not serious | Serious^b^ | None | 25 | NA | 0/25 (0%) | | ⨁◯◯◯  Very low |
| **SAFETY** | | | | | | | | | | | |
| Adverse events (from 10 days post-intervention up to 12 months) | | | | | | | | | | | |
| 2 [54, 55] | Single-arm | Serious^a^ | Not serious | Not serious | Serious^b^ | None | 35 | NA | At 12 m [54]: Oedema: 25/25 (100%)  Pain: 11/25 (44%)  Mild fever: 3/25 (12)  At 10 d [55]:  Minor AEs: 5 | | ⨁◯◯◯  Very low |
| **Serious adverse events** | | | | | | | | | | | |
| 0 | NA | NA | NA | NA | NA | NA | NA | NA | NA | | NA |

*Abbreviations: AE – adverse event, CI – confidence interval, FU– follow-up, HIFU – high-intensity focused ultrasound ablation, HTA – health technology assessment, m – months, NA – not applicable*

*Comments:*

*^a^ According to the HTA-Guidelines and LATITUDES recommended risk of bias tools, single-arm trials are considered to have a high risk of bias.*

*^b^ Small sample size, low number of events.*

*Table A‑17: Evidence profile: Efficacy and safety of laser ablation in early-stage breast cancer*

| **Quality assessment** | | | | | | | **Summary of findings** | | | | | |
| --- | --- | --- | --- | --- | --- | --- | --- | --- | --- | --- | --- | --- |
|  |  |  |  |  |  |  | **Number of patients** | | **Effect** | | **Quality** | |
| **Number  of studies** | **Study design** | **Risk of bias** | **Inconsistency** | **Indirectness** | **Imprecision** | **Other  considerations** | **intervention** | **comparison** | **Relative (95% CI)** | **Absolute (95% CI)** |  |  |
| **EFFICACY** | | | | | | | | | | | | |
| **Overall survival** | | | | | | | | | | | | |
| 0 | NA | NA | NA | NA | NA | NA | NA | NA | NA | | NA | |
| **Disease free survival** | | | | | | | | | | | | |
| 0 | NA | NA | NA | NA | NA | NA | NA | NA | NA | | NA | |
| **Complete tumour ablation/necrosis*** | | | | | | | | | | | | |
| 1 [56] | Single-arm | Serious^a^ | Not serious | Not serious | Serious^b^ | None | 61 | NA | 51/61 (84%) | | ⨁◯◯◯  Very low | |
| **Recurrence/local and distant (follow-up 4 years)** | | | | | | | | | | | | |
| 1 [56] | Single-arm | Serious^a^ | Not serious | Not serious | Serious^b^ | None | 61 | NA | 2/61 (3%) | | ⨁◯◯◯  Very low | |
| **SAFETY** | | | | | | | | | | | | |
| **Adverse events (follow-up 43 months)** | | | | | | | | | | | | |
| 1 [56] | Single-arm | Serious^a^ | Not serious | Not serious | Serious^b^ | None | 61 | NA | Mild AEs: 8  Moderate AEs: 6 | | ⨁◯◯◯  Very low | |
| **Serious adverse events** | | | | | | | | | | | | |
| 0 | NA | NA | NA | NA | NA | NA | NA | NA | NA | | | NA |

*Abbreviations: AE – adverse event, CI – confidence interval, FU– follow-up, LA – laser ablation, m – months, NA – not applicable*

*Comments:*

*^a^ According to the HTA-Guidelines and LATITUDES recommended risk of bias tools, single-arm trials are considered to have a high risk of bias.*

*^b^ Small sample size, low number of events.*

Table A - 18: Excluded studies based on full-text screening with reasons for exclusion

| **Study ID (Author, year, title)** | **Reason for exclusion** |
| --- | --- |
| Dai et al. 2024. Percutaneous microwave ablation: a viable local therapy for breast cancer involving the skin/nipple-areola complex? | Other study design (retrospective) |
| Deckers et al. 2015. Performance analysis of a dedicated breast MR-HIFU system for tumor ablation in breast cancer patients | Other outcomes |
| Fine et al. 2024. ASO Visual Abstract: Cryoablation without Excision for Early-Stage Breast Cancer | Other study design (conference abstract) |
| Gajda et al. 2014. Breast pathology after cryotherapy. Histological regression of breast cancer after cryotherapy | Other study design (retrospective). |
| Haraldsdottir et al. 2015. Long-term Follow-up After Interstitial Laser Thermotherapy of Breast Cancer | Other outcome, other population (later cancer stages) |
| Jimenez et al. 2020. Radiofrequency Ablation of the Surgical Bed After Lumpectomy in Breast-conserving Surgery | Other intervention |
| Kinoshita et al. 2019. RFA experiences, indications and clinical outcomes | Other publication type |
| Kinoshita et al. 2020. Multicenter study to evaluate the efficacy and standardize radiofrequency ablation therapy for small breast carcinomas | Other publication type (abstract) |
| Klimberg et al. 2011. Feasibility of percutaneous excision followed by ablation for local control in breast cancer | Other publication date, other intervention |
| Klimberg et al. 2014. Long-term results of phase II ablation after breast lumpectomy added to extend intraoperative margins (ABLATE l) trial | Other intervention |
| McArthur et al. 2016. A Pilot Study of Preoperative Single-Dose Ipilimumab and/or Cryoablation in Women with Early-Stage Breast Cancer with Comprehensive Immune Profiling | Other intervention |
| Nori et al. 2018. The Evolving Role of Ultrasound Guided Percutaneous Laser Ablation in Elderly Unresectable Breast Cancer Patients: A Feasibility Pilot Study | Other study design (retrospective) |
| Pan et al. 2021. Precision Breast-Conserving Surgery With Microwave Ablation Guidance: A Pilot Single-Center, Prospective Cohort Study | Other intervention |
| Perretta et al. 2021. Ultrasound-Guided Laser Ablation After Excisional Vacuum-Assisted Breast Biopsy for Small Malignant Breast Lesions: Preliminary Results | Other intervention |
| Roca Navarro et al. 2024. Pre-surgical cryoablation in <= 2 cm ER + /HER2-tumors. Prognostic factors for the presence of residual invasive carcinoma | Other outcomes |
| Rubio et al. 2014. Breast-conservative surgery followed by radiofrequency ablation of margins decreases the need for a second surgical procedure for close or positive margins | Other intervention |
| Schwartzberg et al. 2018. Correction to: Phase 2 Open-Label Trial Investigating Percutaneous Laser Ablation for Treatment of Early-Stage Breast Cancer: MRI, Pathology, and Outcome Correlations | Other publication type (correction) |
| Simmons et al. 2014. Long-term results of phase II ablation after breast lumpectomy added to extend intraoperative margins (ABLATE I) trial | Other publication type |
| Takayama et al. 2023. Patients Offer Radiofrequency Ablation Therapy for Early Breast Cancer as Local Therapy (PO-RAFAELO) Study under the Patient-proposed Health Services | Other publication type |
| van de Voort et al. 2023. Comment on: Cryoablation Without Excision for Low-Risk Early-Stage Breast Cancer: 3-Year Interim Analysis of Ipsilateral Breast Tumor Recurrence in the ICE3 Trial | Other publication type (comment) |
| van de Voort et al. 2021. Treatment of early-stage breast cancer with percutaneous thermal ablation, an open-label randomised phase 2 screening trial: rationale and design of the THERMAC trial | Other study design (protocol) |
| Xiao et al. 2024. Thermo-immune synergy: Camrelizumab plus microwave ablation in preoperative early-stage breast cancer | Other study design |
| Zhou et al. 2014. Image and pathological changes after microwave ablation of breast cancer: a pilot study | Other outcomes, other population (later cancer stages) |

Supplementary material B

Search strategies in bibliographic databases

**Cochrane**:

ID Search

#1 MeSH descriptor: [Breast Neoplasms] explode all trees

#2 ((breast* OR mamma*) NEAR (cancer* OR tumo?r* OR carcinom* OR adenom* OR adeno?c* OR sarcoma* OR neoplasm* OR malignan*)) (Word variations have been searched)

#3 #1 OR #2

#4 (thermo?ablat*) (Word variations have been searched)

#5 (thermo-ablat*) (Word variations have been searched)

#6 MeSH descriptor: [Radiofrequency Ablation] explode all trees

#7 (RFA*)

#8 (laser*) (Word variations have been searched)

#9 (PLA):ti,ab,kw

#10 (micro?wave*) (Word variations have been searched)

#11 (micro-wave*) (Word variations have been searched)

#12 MWA*

#13 MeSH descriptor: [Laser Therapy] explode all trees

#14 MeSH descriptor: [Cryotherapy] explode all trees

#15 MeSH descriptor: [Microwaves] explode all trees

#16 #13 OR #14 OR #15

#17 MeSH descriptor: [Ablation Techniques] explode all trees

#18 #16 AND #17

#19 MeSH descriptor: [High-Intensity Focused Ultrasound Ablation] explode all trees

#20 (high-intensity NEXT focus?ed NEXT ultra?sound*) (Word variations have been searched)

#21 (HIFU*) (Word variations have been searched)

#22 MeSH descriptor: [Cryosurgery] explode all trees

#23 (cryo?ablat*) (Word variations have been searched)

#24 (cryo-ablat*) (Word variations have been searched)

#25 ((radio?frequenc* OR radio-frequenc* OR thermal OR thermic OR laser* OR micro?wave* OR micro-wave* OR ultra?sound* OR cryo*) NEAR (ablat* OR irridat* OR hyper?therm* OR hyper-therm* OR hypo?therm* OR hypo-therm*)) (Word variations have been searched)

#26 (Prosense*) (Word variations have been searched)

#27 (Cryocare*) (Word variations have been searched)

#28 (PulsaBlade*) (Word variations have been searched)

#29 (Solero*) (Word variations have been searched)

#30 (AMICA)

#31 (Cool-Tip*) (Word variations have been searched)

#32 (Cool?Tip*) (Word variations have been searched)

#33 #4 OR #5 OR #6 OR #7 OR #8 OR #9 OR #10 OR #11 OR #12 OR #18 OR #19 OR #20 OR #21 OR #22 OR #23 OR #24 OR #25 OR #26 OR #27 OR #28 OR #29 OR #30 OR #31 OR #32

#34 #3 AND #33

#35 #3 AND #33 with Cochrane Library publication date Between Jan 2014 and Jan 2024

#36 #3 AND #33 with Publication Year from 2014 to 2024, in Trials

#37 #35 OR #36

#38 English:la

#39 German:la

#40 #38 OR #39

#41 #37 AND #40

#42 (conference proceeding):pt

#43 (abstract):so

#44 (clinicaltrials OR trialsearch OR ANZCTR OR ensaiosclinicos OR Actrn OR chictr OR cris OR ctri OR registroclinico OR clinicaltrialsregister OR DRKS OR IRCT OR Isrctn OR rctportal OR JapicCTI OR JMACCT OR jRCT OR JPRN OR Nct OR UMIN OR trialregister OR PACTR OR R.B.R.OR REPEC OR SLCTR OR Tcr):so

#45 #42 OR #43 OR #44

#46 #41 NOT #45

122 Hits

**Embase**

Session Results

No. Query Results Results Date

#40. #38 NOT #39 1,059 15 Dec 2024

#39. #38 AND 'Conference Abstract'/it 224 15 Dec 2024

#38. #37 AND [2020-2024]/py AND ([english]/lim OR 1,283 15 Dec 2024

[german]/lim)

#37. #36 AND [2020-2024]/py 1,316 15 Dec 2024

#36. #3 AND #35 3,886 15 Dec 2024

#35. #4 OR #5 OR #6 OR #7 OR #8 OR #9 OR #10 OR #11 OR 244,947 15 Dec 2024

#12 OR #13 OR #14 OR #15 OR #16 OR #17 OR #18 OR

#19 OR #20 OR #21 OR #22 OR #23 OR #24 OR #25 OR

#26 OR #27 OR #28 OR #29 OR #30 OR #31 OR #32 OR

#33 OR #34

#34. 'cool*tip*' 946 15 Dec 2024

#33. 'cool-tip*' 747 15 Dec 2024

#32. 'radiofrequency ablation device'/exp 4,162 15 Dec 2024

#31. 'cool tip rf system'/exp 12 15 Dec 2024

#30. 'cool tip rf ablation system'/exp 14 15 Dec 2024

#29. amica:dn 78 15 Dec 2024

#28. 'amica'/exp 30 15 Dec 2024

#27. solero:dn 25 15 Dec 2024

#26. 'solero'/exp 22 15 Dec 2024

#25. pulsablade* 15 Dec 2024

#24. cryocare:dn 81 15 Dec 2024

#23. 'cryosurgery device'/exp 2,476 15 Dec 2024

#22. 'cryotherapy device'/exp 231 15 Dec 2024

#21. prosense*:dn 21 15 Dec 2024

#20. 'prosense'/exp 17 15 Dec 2024

#19. (radio*frequenc* OR 'radio frequenc*' OR thermal 94,735 15 Dec 2024

OR thermic OR laser* OR micro*wave* OR

'micro-wave*' OR ultra*sound* OR cryo*) NEAR/2

(ablat* OR irridat* OR hyper*therm* OR 'hyper

therm*' OR hypo*therm* OR 'hypo-therm*')

#18. 'cryo-ablat*' 372 15 Dec 2024

#17. cryo*ablat* 15,056 15 Dec 2024

#16. hifu* 9,546 15 Dec 2024

#15. 'high-intensity focus*ed ultra*sound*' 8,668 15 Dec 2024

#14. 'high intensity focused ultrasound'/exp 7,446 15 Dec 2024

#13. mwa* 24,592 15 Dec 2024

#12. 'microwave thermotherapy'/exp 7,667 15 Dec 2024

#11. 'cryoablation'/exp 12,203 15 Dec 2024

#10. 'laser surgery'/exp 75,208 15 Dec 2024

#9. pla:ti,ab 28,879 15 Dec 2024

#8. rfa* 26,679 15 Dec 2024

#7. 'radiofrequency ablation'/exp 50,381 15 Dec 2024

#6. 'thermo-ablat*' 158 15 Dec 2024

#5. thermo*ablat* 1,142 15 Dec 2024

#4. 'thermal ablation'/exp 1,578 15 Dec 2024

#3. #1 OR #2 829,354 15 Dec 2024

#2. (breast* OR mamma*) NEAR/2 (cancer* OR tumo*r* OR 823,436 15 Dec 2024

carcinom* OR adenom* OR adeno*c* OR sarcoma* OR

neoplasm* OR malignan*)

#1. 'breast tumor'/exp 730,379 15 Dec 2024

Ovid **MEDLINE**(R) ALL <1946 to December 17, 2024>

1 exp Breast Neoplasms/ (362254)

2 ((breast* or mamma*) adj3 (cancer* or tumo?r* or carcinom* or adenom* or adeno?c* or sarcoma* or neoplasm* or malignan*)).mp. (530925)

3 1 or 2 (530933)

4 thermo?ablat*.mp. (610)

5 thermo-ablat*.mp. (81)

6 exp Radiofrequency Ablation/ (43823)

7 RFA*.mp. (11281)

8 PLA.mp. (19145)

9 exp Laser Therapy/ (69199)

10 exp Cryotherapy/ (28289)

11 exp Microwaves/ (20709)

12 9 or 10 or 11 (117240)

13 exp Ablation Techniques/ (136295)

14 12 and 13 (64626)

15 MWA*.mp. (3372)

16 exp High-Intensity Focused Ultrasound Ablation/ (2987)

17 high-intensity focus?ed ultra?sound*.mp. (5105)

18 HIFU*.mp. (3433)

19 exp Cryosurgery/ (14579)

20 cryo*ablat*.mp. (5448)

21 cryo-ablat*.mp. (106)

22 ((radio?frequenc* or radio-frequenc* or thermal or thermic or laser* or micro?wave* or micro-wave* or ultra?sound* or cryo*) adj3 (ablat* or irridat* or hyper*therm* or hyper-therm* or hypo?therm* or hypo-therm*)).mp. (53601)

23 Prosense*.mp. (42)

24 Cryocare*.mp. (20)

25 PulsaBlade*.mp. (0)

26 Solero*.mp. (16)

27 AMICA.mp. (92)

28 Cool-Tip*.mp. (136)

29 Cool?Tip*.mp. (7)

30 4 or 5 or 6 or 7 or 8 or 14 or 15 or 16 or 17 or 18 or 19 or 20 or 21 or 22 or 23 or 24 or 25 or 26 or 27 or 28 or 29 (172220)

31 3 and 30 (1866)

32 limit 31 to yr="2014 - 2024" (921)

33 limit 32 to (english or german) (896)

34 remove duplicates from 33 (893)

15.12.2024

**INAHTA**

Search step # Search query,"Hits","Searched At"

30 ((((radiofrequenc* OR radio-frequenc* OR thermal OR thermic OR laser* OR microwave* OR micro-wave* OR ultrasound* OR ultra-sound* OR cryo*) AND (ablat* OR irridat* OR hypertherm* OR hyper-therm* OR hypotherm* OR hypo-therm*)) OR (cryo-ablat*) OR (cryoablat*) OR ("Cryosurgery"[mhe]) OR (HIFU*) OR (high-intensity focussed ultrasound*) OR (high-intensity focused ultrasound*) OR ("High-Intensity Focused Ultrasound Ablation"[mhe]) OR (("Ablation Techniques"[mhe]) AND (("Microwaves"[mhe]) OR ("Laser Therapy"[mhe]) OR ("Cryotherapy"[mhe]))) OR (MWA*) OR (micro-wave*) OR (microwave*) OR (PLA) OR (laser*) OR (RFA*) OR ("Radiofrequency Ablation"[mhe]) OR (thermo-ablat*) OR (thermoablat*)) AND (((breast* OR mamma*) AND (cancer* OR tumor* OR tumour* OR carcinom* OR adenom* OR adenoc* OR adeno-c* OR sarcoma* OR neoplasm* OR malignan*)) OR ("Breast Neoplasms"[mhe]))) FROM 2014 TO 2024,"0","2024-12-15T04:22:51.000000Z"

29 (((radiofrequenc* OR radio-frequenc* OR thermal OR thermic OR laser* OR microwave* OR micro-wave* OR ultrasound* OR ultra-sound* OR cryo*) AND (ablat* OR irridat* OR hypertherm* OR hyper-therm* OR hypotherm* OR hypo-therm*)) OR (cryo-ablat*) OR (cryoablat*) OR ("Cryosurgery"[mhe]) OR (HIFU*) OR (high-intensity focussed ultrasound*) OR (high-intensity focused ultrasound*) OR ("High-Intensity Focused Ultrasound Ablation"[mhe]) OR (("Ablation Techniques"[mhe]) AND (("Microwaves"[mhe]) OR ("Laser Therapy"[mhe]) OR ("Cryotherapy"[mhe]))) OR (MWA*) OR (micro-wave*) OR (microwave*) OR (PLA) OR (laser*) OR (RFA*) OR ("Radiofrequency Ablation"[mhe]) OR (thermo-ablat*) OR (thermoablat*)) AND (((breast* OR mamma*) AND (cancer* OR tumor* OR tumour* OR carcinom* OR adenom* OR adenoc* OR adeno-c* OR sarcoma* OR neoplasm* OR malignan*)) OR ("Breast Neoplasms"[mhe])),"15","2024-12-15T04:22:37.000000Z"

28 (((radiofrequenc* OR radio-frequenc* OR thermal OR thermic OR laser* OR microwave* OR micro-wave* OR ultrasound* OR ultra-sound* OR cryo*) AND (ablat* OR irridat* OR hypertherm* OR hyper-therm* OR hypotherm* OR hypo-therm*)) OR (cryo-ablat*) OR (cryoablat*) OR ("Cryosurgery"[mhe]) OR (HIFU*) OR (high-intensity focussed ultrasound*) OR (high-intensity focused ultrasound*) OR ("High-Intensity Focused Ultrasound Ablation"[mhe]) OR (("Ablation Techniques"[mhe]) AND (("Microwaves"[mhe]) OR ("Laser Therapy"[mhe]) OR ("Cryotherapy"[mhe]))) OR (MWA*) OR (micro-wave*) OR (microwave*) OR (PLA) OR (laser*) OR (RFA*) OR ("Radiofrequency Ablation"[mhe]) OR (thermo-ablat*) OR (thermoablat*)) AND (((breast* OR mamma*) AND (cancer* OR tumor* OR tumour* OR carcinom* OR adenom* OR adenoc* OR adeno-c* OR sarcoma* OR neoplasm* OR malignan*)) OR ("Breast Neoplasms"[mhe])),"15","2024-12-15T04:20:24.000000Z"

27 ((radiofrequenc* OR radio-frequenc* OR thermal OR thermic OR laser* OR microwave* OR micro-wave* OR ultrasound* OR ultra-sound* OR cryo*) AND (ablat* OR irridat* OR hypertherm* OR hyper-therm* OR hypotherm* OR hypo-therm*)) OR (cryo-ablat*) OR (cryoablat*) OR ("Cryosurgery"[mhe]) OR (HIFU*) OR (high-intensity focussed ultrasound*) OR (high-intensity focused ultrasound*) OR ("High-Intensity Focused Ultrasound Ablation"[mhe]) OR (("Ablation Techniques"[mhe]) AND (("Microwaves"[mhe]) OR ("Laser Therapy"[mhe]) OR ("Cryotherapy"[mhe]))) OR (MWA*) OR (micro-wave*) OR (microwave*) OR (PLA) OR (laser*) OR (RFA*) OR ("Radiofrequency Ablation"[mhe]) OR (thermo-ablat*) OR (thermoablat*),"795","2024-12-15T04:19:42.000000Z"

26 (radiofrequenc* OR radio-frequenc* OR thermal OR thermic OR laser* OR microwave* OR micro-wave* OR ultrasound* OR ultra-sound* OR cryo*) AND (ablat* OR irridat* OR hypertherm* OR hyper-therm* OR hypotherm* OR hypo-therm*),"301","2024-12-15T04:17:05.000000Z"

25 cryo-ablat*,"0","2024-12-15T04:13:31.000000Z"

24 cryoablat*,"35","2024-12-15T04:13:19.000000Z"

23 "Cryosurgery"[mhe],"45","2024-12-15T04:13:01.000000Z"

22 HIFU*,"27","2024-12-15T04:12:40.000000Z"

21 high-intensity focussed ultrasound*,"3","2024-12-15T04:11:45.000000Z"

20 high-intensity focused ultrasound*,"63","2024-12-15T04:11:32.000000Z"

19 "High-Intensity Focused Ultrasound Ablation"[mhe],"54","2024-12-15T04:11:03.000000Z"

18 ("Ablation Techniques"[mhe]) AND (("Microwaves"[mhe]) OR ("Laser Therapy"[mhe]) OR ("Cryotherapy"[mhe])),"228","2024-12-15T04:10:11.000000Z"

17 "Ablation Techniques"[mhe],"564","2024-12-15T04:10:03.000000Z"

16 ("Microwaves"[mhe]) OR ("Laser Therapy"[mhe]) OR ("Cryotherapy"[mhe]),"304","2024-12-15T04:09:40.000000Z"

15 "Microwaves"[mhe],"28","2024-12-15T04:09:00.000000Z"

14 "Laser Therapy"[mhe],"218","2024-12-15T04:08:14.000000Z"

13 "Cryotherapy"[mhe],"59","2024-12-15T04:07:48.000000Z"

12 MWA*,"6","2024-12-15T04:06:52.000000Z"

11 micro-wave*,"0","2024-12-15T04:06:29.000000Z"

10 microwave*,"53","2024-12-15T04:06:24.000000Z"

9 PLA,"3","2024-12-15T04:05:54.000000Z"

8 laser*,"347","2024-12-15T04:04:50.000000Z"

7 RFA*,"70","2024-12-15T04:04:19.000000Z"

6 "Radiofrequency Ablation"[mhe],"238","2024-12-15T04:03:46.000000Z"

5 thermo-ablat*,"0","2024-12-15T04:03:09.000000Z"

4 thermoablat*,"3","2024-12-15T04:03:04.000000Z"

3 ((breast* OR mamma*) AND (cancer* OR tumor* OR tumour* OR carcinom* OR adenom* OR adenoc* OR adeno-c* OR sarcoma* OR neoplasm* OR malignan*)) OR ("Breast Neoplasms"[mhe]),"929","2024-12-15T04:02:32.000000Z"

2 (breast* OR mamma*) AND (cancer* OR tumor* OR tumour* OR carcinom* OR adenom* OR adenoc* OR adeno-c* OR sarcoma* OR neoplasm* OR malignan*),"867","2024-12-15T04:02:23.000000Z"

1 "Breast Neoplasms"[mhe],"708","2024-12-15T03:59:17.000000Z"

Total hits 0

Date of search 15.12.2024

**Update Search in Pubmed**

Date of search: 01.05.2026

Search terms: (("Breast Neoplasms"[Mesh] OR (breast*[tiab] OR mamma*[tiab]) AND (cancer*[tiab] OR tumor*[tiab] OR tumour*[tiab] OR carcinoma*[tiab] OR adenoma*[tiab] OR neoplasm*[tiab] OR malignan*[tiab])) AND ( "Radiofrequency Ablation"[Mesh] OR "Cryotherapy"[Mesh] OR "Microwaves"[Mesh] OR "Laser Therapy"[Mesh] OR "High-Intensity Focused Ultrasound Ablation"[Mesh] OR "Ablation Techniques"[Mesh] OR thermoablat*[tiab] OR thermo-ablat*[tiab] OR RFA[tiab] OR MWA[tiab] OR HIFU[tiab] OR cryoablat*[tiab] OR cryo-ablat*[tiab] OR "high-intensity focused ultrasound"[tiab] OR (radiofrequency OR radio-frequency OR thermal OR thermic OR laser OR microwave* OR micro-wave* OR ultrasound* OR cryo*) AND (ablat* OR irradiat* OR hypertherm* OR hypotherm*) OR ProSense[tiab] OR Cryocare[tiab] OR PusaBlade[tiab] OR Solero*[tiab] OR AMICA[tiab] OR "Cool-Tip"[tiab] OR CoolTip[tiab] )) Filters: Abstract, Clinical Study, Clinical Trial, Clinical Trial, Phase I, Clinical Trial, Phase II, Clinical Trial, Phase III, Clinical Trial, Phase IV, Comparative Study, Controlled Clinical Trial, Evidence Synthesis, Meta-Analysis, Observational Study, Pragmatic Clinical Trial, Randomized Controlled Trial, Scoping Review, Systematic Review, Validation Study, from 2024 - 2026

Search strategies in clinical trials registries

Date of search: 09.01.2025

**ClinicalTrials.gov**:

Breast Cancer OR Breast Neoplasms OR Breast Carcinoma in Situ OR Breast Tumour OR Breast Tumor OR Breast Tumors OR Breast Carcinoma OR Breast Adenocarcinoma OR Breast Sarcoma OR Breast Malignant Tumor OR Mammary Cancer OR Mammary Neoplasms, Human OR Mammary Tumor OR Mamma Carcinoma in Condition/disease

ablation OR thermoablation OR Microwave ablation OR laser ablation OR Laser ablation thermal therapy OR Cryoablation OR RFA OR PLA OR MWA OR HIFU OR PROSENSE™ OR Cryocare OR PulsaBlade OR Solero OR AMICA OR Cool-tip RF Ablation System OR Cool-tip electrode for Radiofrequency Ablation in Intervention/treatment

Last update posted from 01/01/2014 to 12/31/2024

98 studies identified

**WHO-ICTRP** (Advanced Search mode):

Breast Cancer OR Breast Neoplasms OR Breast Carcinoma OR Breast Tumour OR Breast Tumor OR Breast Adenocarcinoma OR Breast Sarcoma OR Breast Malignant Tumor OR Mammary Cancer OR Mammary Neoplasms OR Mammary Tumor OR Mammary Tumour OR Mamma Carcinoma in the Condition

ablation OR thermoablation OR Microwave ablation OR laser ablation OR Cryoablation OR RFA OR PLA OR MWA OR HIFU OR PROSENSE OR Cryocare OR PulsaBlade OR Solero OR AMICA OR Cool-tip in the Intervention

Date of registration is between 01/01/2014 and 31/12/2024

51 (21 further) studies identified

**EU Clinical Trials (EUdraCT) Register** (Advanced Search mode):

Search string: (Breast Cancer OR Breast Neoplasms OR Breast Carcinoma OR Breast Tumour OR Breast Tumor OR Breast Adenocarcinoma OR Breast Sarcoma OR Breast Malignant Tumor OR Mammary Cancer OR Mammary Neoplasms OR Mammary Tumor OR Mammary Tumour OR Mamma Carcinoma) AND (ablation OR thermoablation OR Microwave ablation OR laser ablation OR Cryoablation OR RFA OR PLA OR MWA OR HIFU OR PROSENSE OR Cryocare OR PulsaBlade OR Solero OR AMICA OR Cool-tip)

Selected Date Range: 2014-01-01 to 2024-12-31

11 (0 relevant) studies identified

Supplementary material C

Table A - 19: List of ongoing trials

| **Technology** | **Trial identifier** | **Trial name** | **Trial type** |
| --- | --- | --- | --- |
| CYA | NCT05505643 | Cryoablation vs Lumpectomy in T1 Breast Cancers | RCT |
| CYA | ChiCTR2100051677 | Clinical trial on comparing the effectiveness of co-ablation and breast-conserving surgery in treating patients with low-risk early breast cancer | NRSI |
| CYA | NCT05972343 | COOL-IT-PRO: Cryoablation of Breast Cancer in the Elderly | Single-arm |
| CYA | NCT05398497 | FreezIng bReaST Cancer in Brazil: a Before-after Cohort Study | Single-arm |
| CYA | NCT06387173 | Registry of Patients Undergoing Cryoablation for Breast Tumors | Single-arm |
| CYA | NCT06300125 | Percutaneous Cryoablation of Low-risk Early Breast Cancer | Single-arm |
| CYA | NCT05218044 | Cryoablation as a Minimally Invasive Alternative Treatment for Early-stage Breast Cancer | Single-arm |
| CYA | NCT04334785 | Evaluation for the Effectiveness and Safety of Cryoablation in Stage I Breast Cancer | Single-arm |
| CYA | NCT02576106 | Evaluation of Percutaneous Treatment by Cryoablation in Breast Cancer | Single-arm |
| CYA | ChiCTR2300076716 | Low-risk Early Breast Cancer Cryoablation Study | Single-arm |
| MWA | NCT04626986 | Comparison of Microwave Ablation With Breast Conserving Surgery in Early-stage Breast Cancer | NRSI |
| MWA | NCT06288620 | Microwave Ablation in the Treatment of Early-stage Breast Cancer | Single-arm |
| MWA | NCT05887154 | Non-Surgical Treatment (Microwave Ablation) of Breast Cancer | Single-arm |
| MWA | ChiCTR2000029155 | Percutaneous microwave ablation of small breast cancer | Single-arm |
| MWA | ChiCTR1900023959 | Percutaneous microwave ablation of small breast cancer | Single-arm |
| MWA | ChiCTR1800015984 | MR-guided microwave ablation for breast cancer and Evaluation of MRI ablation | Single-arm |
| MWA | NL-OMON51501 | Minimal invasive Microwave Ablation in early stage breaST cancER, a feasibility study | Single-arm |
| RFA | NCT04389216 | Radiofrequency Ablation (RFA) in Breast Tumors | Single-arm |
| RFA | ChiCTR2000029665 | Surgery Combined With Radiofrequency Ablation in Breast Cancer | Single-arm |
| RFA | JPRN-UMIN000020805 | Phase II study on radiofrequency ablation in early breast cancer | Single-arm |
| RFA | JPRN-UMIN000013836 | Phase II study on radiofrequency ablation in stage 0 and I breast cancer without extensive intraductal components | Single-arm |
| LA | NCT03463954 | Confirmatory Clinical Evaluation of Novilase® Laser Therapy System for Ablation of Breast Fibroadenomas | Single-arm |
| HIFU | NCT05350059 | The HIFUB Study (HIFU in Breast Cancer) | Single-arm |
| HIFU | NCT03342625 | High Intensity Focused Ultrasound Treatment of Early-stage Breast Cancer | Single-arm |
| HIFU | NCT01620359 | Study of ExAblate Focused Ultrasound Ablation Treatment for Breast Cancer | Single-arm |

*Abbreviations: CYA – cryoablation, HIFU – high-intensity focused ultrasound, LA – laser ablation, MWA – microwave ablation, NRSI – non-randomised study of intervention, RCT – randomised controlled trial, RFA – radiofrequency ablation*

1. Event numbers calculated by the review authors from percentages of the study publication. [↑](#footnote-ref-1)
